# Transepidermal Delivery of Calcium Hydroxyapatite (CaHA) Microneedling: A Novel Approach for Inducing Collagen Types III and IV

**DOI:** 10.3390/biomedicines13102463

**Published:** 2025-10-10

**Authors:** Andréia Luiza Oliveira Costa, Fernando Veloso Caldeira Barcellos, Maria Tereza Scardua Mariano, Eloá Mangabeira Santos, Lorena dos Reis Pereira Queiroz, Erivelton Pereira Santos, Vitória Regina Oliveira de Melo, Sérgio Henrique Sousa Santos, Lucyana Conceição Farias, Alfredo Maurício Batista de Paula, André Luiz Sena Guimarães

**Affiliations:** 1 Department of Dentistry, Universidade Estadual de Montes, Montes Claros 39408-354, MG, Brazil; andreialocosta@hotmail.com (A.L.O.C.); fernandovelosocaldeira@gmail.com (F.V.C.B.); esmangabeira@gmail.com (E.M.S.); lorenadosreis31@gmail.com (L.d.R.P.Q.); eriveltonbiomedicina@hotmail.com (E.P.S.); vitoriaom3@gmail.com (V.R.O.d.M.); lucyana.farias@unimontes.br (L.C.F.); ambpatologi@gmail.com (A.M.B.d.P.); 2 Tereza Scardua Institute São Paulo, São Paulo 04711-030, SP, Brazil; maria@terezascardua.com.br; 3 Institute of Agricultural Sciences, Universidade Federal de Minas Gerais, Montes Claros 30130-100, MG, Brazil; sergiosousas@hotmail.com

**Keywords:** microneedling, transepidermal delivery, calcium hydroxyapatite (CaHA), extracellular matrix remodeling, collagen expression, hyaluronidase, keratinocytes, ex vivo skin model

## Abstract

**Background/Objectives:** Microneedling is increasingly applied to enhance transepidermal drug delivery. Calcium hydroxyapatite (CaHA) has recognized biostimulatory properties, but its delivery and distribution within the dermis remain poorly characterized. This study aimed to evaluate the efficacy of microneedling for transepidermal CaHA delivery, focusing on retention, dispersion, and collagen expression, and to investigate the role of hyaluronidase in modulating these effects. **Methods:** Fluorescently labeled CaHA nanopowder was characterized using scanning electron microscopy (SEM) and confocal fluorescence microscopy. Nine experimental groups were established, varying microneedling intensity and hyaluronidase application. Ex vivo porcine skin samples were analyzed histologically for CaHA penetration and distribution. HaCaT keratinocyte cultures were treated with CaHA, and mRNA expression of collagen types I, III, IV, and MMP-1 was quantified. **Results:** High-intensity microneedling improved CaHA retention but caused tissue damage. Low-intensity microneedling showed limited retention, enhanced by hyaluronidase co-application. In vitro, CaHA increased collagen type III and IV expression, while collagen type I and MMP-1 remained unchanged. **Conclusions:** Microneedling effectively delivers CaHA into the dermis and stimulates collagen remodeling. Optimizing microneedling intensity is essential to balance efficacy and safety, and hyaluronidase may enhance clinical outcomes by improving CaHA distribution.

## 1. Introduction

Microneedling has gained widespread recognition as an effective transdermal drug delivery technique, offering minimal invasiveness while enhancing the penetration of bioactive compounds into the skin [1]. This technique involves the creation of microchannels in the epidermis and dermis, facilitating the absorption of therapeutic agents while simultaneously stimulating collagen production and skin regeneration [2]. Among the materials utilized for regenerative purposes, calcium hydroxyapatite (CaHA) has been extensively studied for its biocompatibility [3], ability to promote neocollagenesis [4], and role in soft tissue augmentation [5]. Recent investigation [6] into the biostimulatory effects of CaHA suggests that the material provides structural support and induces long-term collagen synthesis, thereby contributing to sustained skin rejuvenation.

The extracellular matrix (ECM) is pivotal in maintaining skin integrity, with key components such as collagen type I and III [7], elastin [8], and glycosaminoglycans contributing to biomechanical properties. Aging, ultraviolet radiation, and environmental factors lead to ECM degradation, resulting in loss of skin elasticity, hydration, and overall structural integrity [9]. Regenerative techniques such as microneedling-mediated delivery of regenerative actives have emerged as a promising strategy [1]. Studies have demonstrated that CaHA stimulates fibroblast proliferation and promotes the deposition of new collagen fibers [10], with long-lasting effects surpassing traditional hyaluronic acid fillers [11]. Furthermore, comparative studies indicate that CaHA-based fillers, particularly those suspended in carboxymethyl cellulose (CMC), exhibit superior rheological properties, enhanced biostimulation, and improved integration into soft tissues, making them ideal candidates for aesthetic and therapeutic applications [12].

Keratinocytes, the predominant cell type in the epidermis, play a crucial role in modulating the skin’s barrier function and responding to external stimuli [13]. The ability of microneedling to facilitate the direct delivery of CaHA to keratinocytes and deeper dermal layers enhances its therapeutic potential, ensuring efficient cellular uptake and activation of regenerative pathways [14]. In addition to their barrier function, keratinocytes have been shown to actively participate in ECM remodeling by producing collagen, particularly type IV, which contributes to the structural integrity of the basement membrane and supports dermal-epidermal interactions [15].

Despite growing evidence supporting microneedling as a method for CaHA delivery, further research is needed to refine treatment protocols, assess long-term safety, and better understand its biostimulatory mechanisms. This study aims to evaluate the effectiveness of microneedling in delivering CaHA into the dermis by examining its penetration, tissue distribution, and biostimulatory effects. Additionally, it investigates the role of hyaluronidase in enhancing CaHA dispersion and retention and the potential benefits of combination therapies for optimizing ECM remodeling and collagen synthesis in an ex vivo skin model.

## 2. Materials and Methods

### 2.1. Ethical Approval

**Figure 1:**
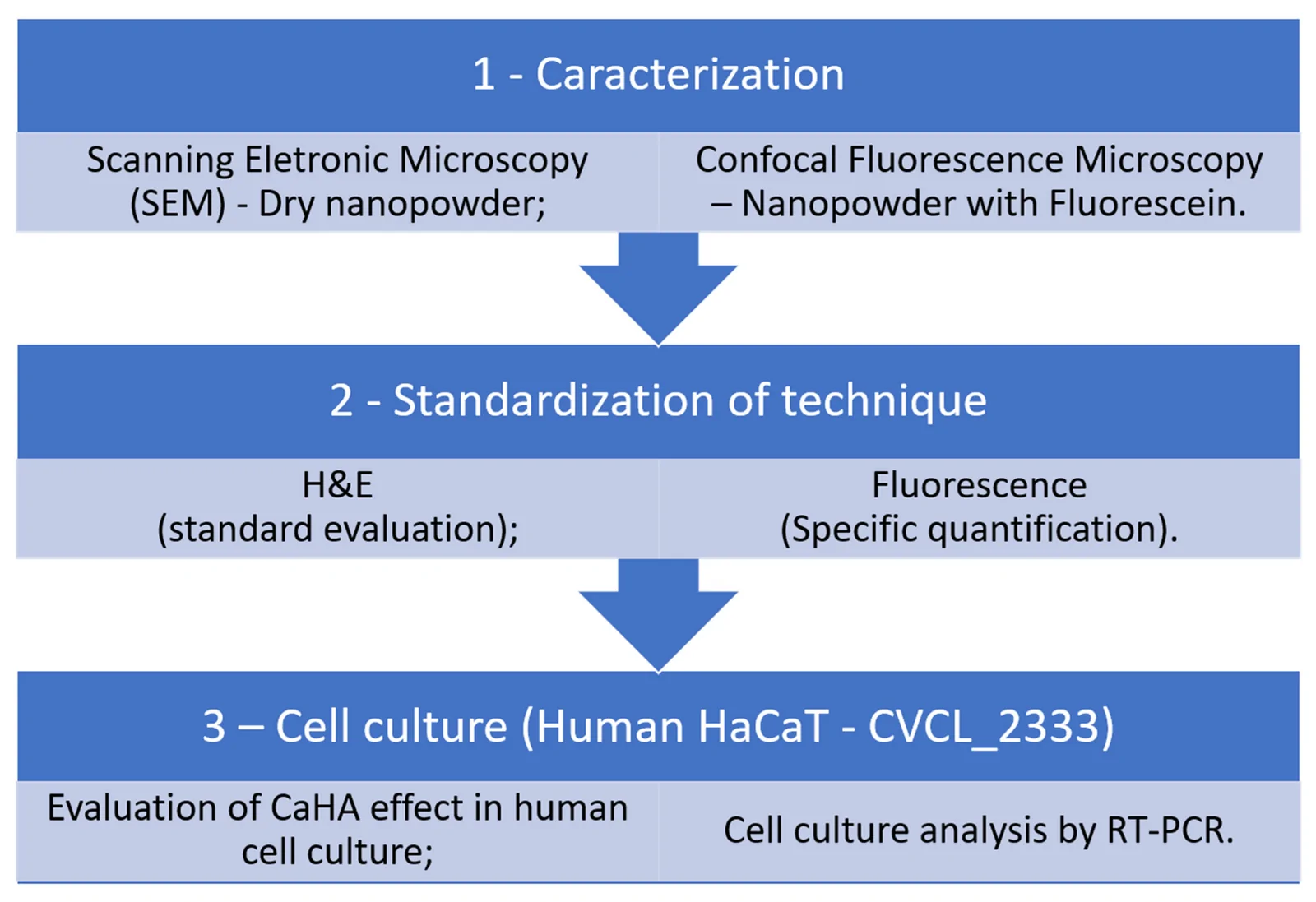
A schematic representation of the experimental workflow is depicted. The first step consisted of particle characterization by scanning electron microscopy (SEM). As the formulation to be delivered by microneedling was in liquid suspension, dispersion and morphology were further analyzed by confocal microscopy to establish the most suitable protocol. Nine experimental groups were initially evaluated in hematoxylin and eosin (H&E) staining, followed by fluorescence microscopy to confirm specific product distribution. Fluorescence analysis provided an advantage over H&E, as it selectively detected the labeled particles, reducing nonspecific signals. Finally, functional validation was performed using epithelial cell cultures, the primary biological target of the proposed treatment.

Institutional Review Board Statement: This study did not involve live animals. When animal-derived tissues were used, they were obtained from slaughterhouse by-products intended for human consumption. Following Brazilian federal law (Law No. 11.794/2008), their use does not constitute animal experimentation and does not require ethics approval. Cell culture experiments utilized the immortalized human keratinocyte cell line HaCaT, acquired from the Banco de Células do Rio de Janeiro (BCRJ, catalog number 0341). This cell line is registered in the Cellosaurus database (accession CVCL_0038, RRID: CVCL_0038). The study was approved by the Research Ethics Committee of Universidade Estadual de Montes Claros (CEP/Unimontes), under protocol CAAE 52760016.9.0000.5146. The experimental procedure was performed according to the protocol illustrated in Figure 1.

### 2.2. Preparation of CaHA and Hyaluronidase

The CaHA nanopowder (Victalab, São Paulo, Brazil) used in this study is characterized as spherical, with an average particle size of approximately 160 nm. The manufacturer did not specify the molecular weight. The material has a density of 1.67 g/cm^3^ and a purity of ≥97%, with a zeta potential of −10 mV and a pH of 7.36. CaHA nanopowder was dispersed in a 3% polyvinylpyrrolidone (PVP; Sigma-Aldrich, St. Louis, MO, USA) solution (3 g PVP in 100 g distilled water). For uniform distribution, 2 g of CaHA was mixed with 20 mL of this solution under constant agitation. A hyaluronidase solution (0.1 mL) was prepared with 500 units of turbidity reduction (UTR) hyaluronidase (Sigma-Aldrich, St. Louis, MO, USA), 5 mg of adenosine triphosphate (ATP; Sigma-Aldrich), and 1000 IU of catalase (Sigma-Aldrich) in 4 mL of reconstituted stock solution to enhance tissue permeability.

#### Fluorescent Labeling of Calcium Hydroxyapatite

To enable fluorescence imaging, CaHA was labeled with a 1% fluorescein solution (Fludiag™ 1% Solução Oftálmica, 3 mL; Latinofarma, São Paulo, Brazil). The stained product was washed five times in distilled water to remove excess dye and reconstituted in distilled water for use in experiments.

### 2.3. Morphological and Structural Analysis

Scanning electron microscopy (SEM): CaHA morphology was visualized using a Prism E Scanning Electron Microscope (Thermo Fisher Scientific, Waltham, MA, USA) at 30 kV with a 4.0 spot size. Samples were affixed on aluminum stubs with carbon tape and imaged using an Everhart–Thornley secondary electron detector.

Confocal fluorescence microscopy: Labeled CaHA samples were imaged using a Zeiss LSM 980 Confocal Fluorescence Microscope (Carl Zeiss AG, Oberkochen, Germany) with Alexa Fluor 488 excitation, under objective lenses ranging from 5× to 40× magnification. Controls were imaged with enhanced intensity for contrast. Images were saved in TIFF format with minimal editing.

### 2.4. Transepidermal Delivery Protocol

The procedure began with the topical application of 0.05 mL of hyaluronidase (Victalab, São Paulo, Brazil), when applicable, or the corresponding control solution, followed by a one-minute absorption period. Microneedling was then performed using a motorized microneedling pen (Tinex Pen, ARTELE Harmonização Facial Ltd.a., São Paulo, Brazil) fitted with an Aurora Gen III cartridge (1249 CM, Dragoart, Hangzhou, Zhejiang, China), equipped with 49 stainless steel needles (1.5 mm length, 0.35 mm diameter) arranged in two slightly curved rows to enhance skin contact during application as described before [16]. The device operates via a vibration/motorized mechanism, enabling precise control of puncture depth and frequency, and was calibrated to a penetration depth of approximately 0.4 mm. In all cases, only light pressure was applied to the tissue surface. For the study groups in which enzymatic pretreatment was employed to enhance dermal permeability, the CaHA formulation was supplemented with 0.1 mL of an enzymatic mixture composed of 500 units of turbidity reduction (UTR) hyaluronidase, 5 mg adenosine triphosphate (ATP), and 1000 IU catalase in 4 mL of diluent before application. When applicable, a second 0.05 mL dose of hyaluronidase was manually spread over the same area. Subsequently, 0.05 mL of the CaHA (Victalab, São Paulo, Brazil) used formulation was applied topically and allowed to air-dry, followed by a final 0.1 mL dose (previously vortexed) administered in two 0.05 mL fractions with a five-minute interval between applications. For vigorous microneedling, the procedure was carried out continuously for 1 min over the treatment area to ensure uniform coverage. For light microneedling, only 10 passes were performed over the treatment area [16].

### 2.5. Experimental Design

Nine groups were evaluated to test CaHA application methods and the role of hyaluronidase:

- Group 1: Topical CaHA and intense microneedling (1 min), no hyaluronidase.
- Group 2: Same as Group 1 and topical hyaluronidase.
- Group 3: CaHA aspirated into microneedling pen and intense microneedling.
- Group 4: Same as Group 3 and topical hyaluronidase.
- Group 5: Light microneedling with CaHA-loaded pen.
- Group 6: Hyaluronidase injected, then light microneedling with CaHA.
- Group 7: Topical hyaluronidase before light microneedling with CaHA.
- Group 8: Negative control (no treatment).
- Group 9: Positive control (direct injection of CaHA, no hyaluronidase).

### 2.6. Ex Vivo Skin Permeation Analysis

Porcine skin was selected as an ex vivo model to simulate human skin for the assessment of CaHA (Victalab, São Paulo, Brazil) penetration. Samples were fixed in 10% neutral-buffered formalin (Ciavicco, São Paulo, SP, Brazil), dehydrated through a graded ethanol series (Dinâmica, Indaiatuba, SP, Brazil), cleared in xylene (Vetec, Duque de Caxias, RJ, Brazil), and embedded in paraffin (Synth, Diadema, SP, Brazil) [17]. Tissue sections (3 μm thick) were obtained using a microtome (American Optical Spencer 820, Buffalo, NY, USA) and stained with hematoxylin and eosin (Vetec, Duque de Caxias, RJ, Brazil) for histological evaluation [18]. Slides were scanned using a MoticEasyScan Infinity 60 digital slide scanner (Motic, Hong Kong, China). For fluorescence analysis, sections containing fluorescein-labeled (Fludiag™ 1% Solução Oftálmica, 3 mL; Latinofarma, São Paulo, Brazil) CaHA were mounted with an antifade mounting medium (ProLong™ Gold Antifade Mountant, Thermo Fisher Scientific, Waltham, MA, USA) and imaged using appropriate excitation and emission filter settings to ensure optimal visualization of the labeled particles.

#### Quantification of CaHA Distribution

Quantitative image analysis was performed using ImageJ/Fiji software (National Institutes of Health, Bethesda, MD, USA; version not specified, accessed on 10 September 2025). [19]. Images were converted to 8-bit grayscale, and thresholds were set at 0–135 for H&E and 14–121 for fluorescence images [20]. Data from ten fields per sample were analyzed to ensure consistency.

### 2.7. Cell Culture

The immortalized human keratinocyte HaCaT cell line was obtained from the Rio de Janeiro Cell Bank (Banco de Células do Rio de Janeiro, Rio de Janeiro, RJ, Brazil), which provides quality certificates including short tandem repeat (STR) authentication and mycoplasma-free certification. Cells were cultured in Dulbecco’s Modified Eagle’s Medium/F12 (DMEM/F12; Gibco, Billings, MT, USA) supplemented with 10% fetal bovine serum (FBS; Gibco, Billings, MT, USA), 400 ng/mL hydrocortisone (Sigma-Aldrich, St. Louis, MO, USA), and 1% antibiotic/antimycotic solution (Invitrogen, Carlsbad, CA, USA). Passage numbers were restricted to 30–32 to maintain phenotypic stability. Cultures were maintained at 37 °C in a humidified atmosphere containing 5% CO_2_ under standardized conditions (Forma™ Series 3 Water Jacketed CO_2_ Incubator, Thermo Scientific, Waltham, MA, USA). For experiments, cells were seeded in six-well plates (Corning Inc., Corning, NY, USA) at a density of 3 × 10^5^ cells per well [21]. Treatments were initiated once cultures had reached a minimum of 80% confluence. To synchronize the cell cycle, cells were subjected to 24 h of complete serum starvation before treatment. Each experiment included at least three independent biological replicates, and data collection and outcome assessment were performed in a blinded fashion. All assays were validated to ensure reproducibility, and results are reported in full according to predefined outcome measures.

### 2.8. Expression by Real-Time PCR

Total RNA was extracted from dorsal skin tissues using TRIzol™ Reagent (Thermo Fisher Scientific, Waltham, MA, USA), and residual genomic DNA was removed using Amplification Grade DNase I (Invitrogen, Carlsbad, CA, USA) as previously described [22]. Gene expression analysis was performed using validated TaqMan^®^ Gene Expression Assays (Thermo Fisher Scientific, Waltham, MA, USA) for COL1A1 (Hs00164004_m1), COL2A1 (Hs01060345_m1), COL4A1 (Hs00266237_m1), and MMP1 (Hs00899658_m1). The endogenous control was GAPDH (Hs02758991_g1). Reactions were conducted in 384-well plates using FAM™-MGB probes, with amplification performed on a QuantStudio™ 6 Real-Time PCR System (Thermo Fisher Scientific, Waltham, MA, USA) under standard cycling conditions. All reactions were run in duplicate, and relative gene expression levels were calculated using the 2^−ΔΔCt^ method.

### 2.9. Statistical Analysis

All statistical analyses were conducted using GraphPad Prism, version 8.0 (GraphPad Software, Inc., San Diego, CA, USA). Data distribution was assessed with the Kolmogorov–Smirnov and Shapiro–Wilk normality tests. For normally distributed data, parametric analyses were applied, while nonparametric tests were used when assumptions of normality were not met. Comparisons among multiple groups were performed using one-way ANOVA followed by Tukey’s post hoc test (parametric) or Kruskal–Wallis test followed by Dunn’s multiple comparison test (nonparametric). When only two groups were compared, Student’s *t*-test (parametric) or Mann–Whitney U test with Bonferroni correction (nonparametric) was applied. Results are reported as mean ± standard deviation (SD), and statistical significance was defined as *p* < 0.05.

## 3. Results

### 3.1. CaHA Morphology

**Figure 2:**
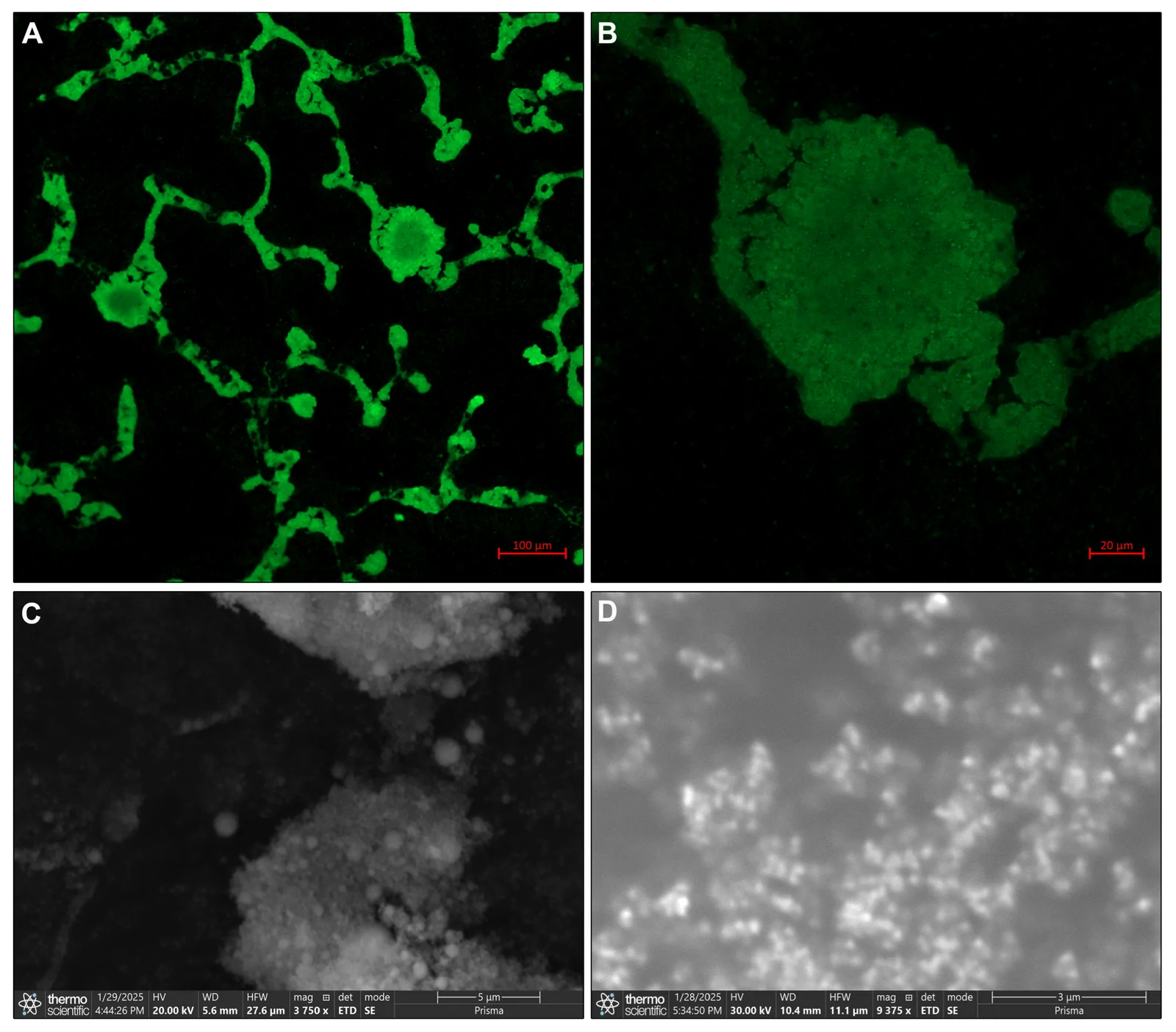
Characterization of CaHA particles by confocal fluorescence microscopy and scanning electron microscopy (SEM). CaHA was prepared by diluting 0.8 g of CaHA and 0.8 g of PVP in 1% sodium fluorescein solution. (**A**,**B**) Confocal fluorescence images acquired on a Zeiss LSM 980, showing CaHA + PVP + fluorescein at magnifications of 10× (0.3 μm objective) (scale bar = 100 μm) and 40× (1.2 μm objective) (scale bar = 20 μm), respectively. The Alexa Fluor 488 laser was used, with a wavelength of 517 nm. (**C**,**D**) SEM images obtained using a Thermo Fisher Prism E scanning electron microscope in High Vacuum mode with an Everhart-Thornley Detector (ETD) demonstrating the nanoparticle and porous morphology of CaHA at 3750× (scale bar = 5 μm) and 9375× (scale bar = 3 μm).

Confocal fluorescence microscopy revealed a uniform distribution of CaHA within the PVP matrix at both 10× and 40× magnifications. SEM analysis confirmed the particulate and porous morphology of CaHA, supporting its potential for tissue interaction and biostimulatory effects (Figure 2; Supplementary Figure S14).

### 3.2. Histological Insights

**Figure 3:**
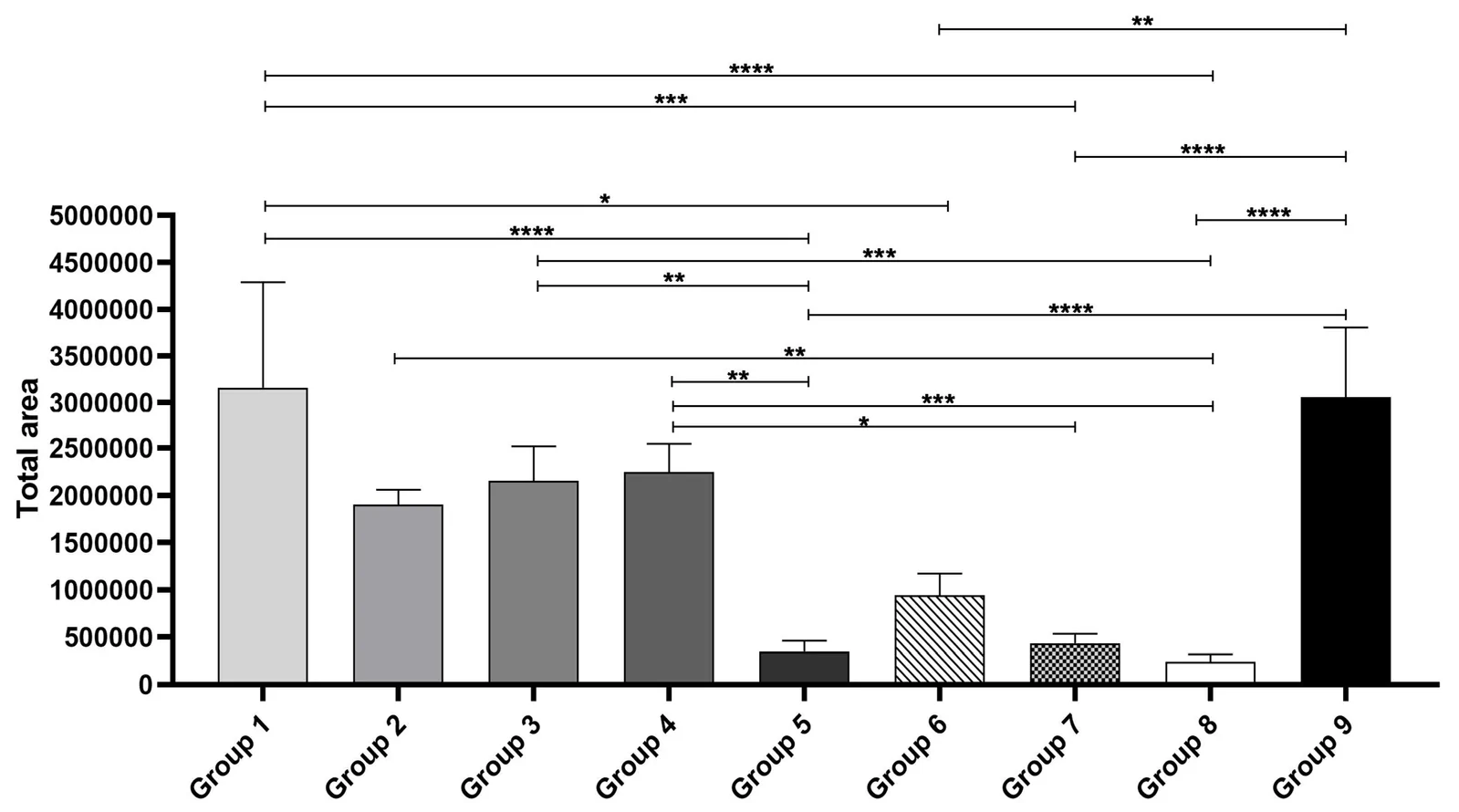
Statistical analyses of CaHA retention in new experimental groups using Graphpad PRISMA software version 8.0: G1, topical CaHA + intense microneedling; G2, same as G1 + topical hyaluronidase; G3, CaHA aspirated into the microneedling pen + intense microneedling; G4, same as G3 + topical hyaluronidase; G5, light microneedling with CaHA pen; G6, hyaluronidase injection followed by light microneedling with CaHA; G7, topical hyaluronidase before light microneedling with CaHA; G8, negative control (no treatment); and G9, positive control (direct CaHA injection without hyaluronidase). Quantitative analysis showed that groups G1–G4 had approximately 2.5–3.0-fold higher CaHA retention compared to light microneedling groups (G5–G7). Data are shown as mean ± SD of three independent experiments. * *p* < 0.01; ** *p* < 0.001; *** *p* < 0.0001; **** *p* < 0.00001.

Histological examination demonstrated distinct differences in CaHA retention across treatment groups. Intense microneedling, whether performed with topical application (Supplementary Figures S1 and S2) or direct aspiration into the microneedling pen (Supplementary Figures S3 and S4), promoted substantial dermal CaHA deposition. The addition of hyaluronidase did not significantly alter outcomes in these high-intensity protocols, indicating that mechanical disruption from microneedling was the primary factor facilitating penetration (Figure 3).

By contrast, low-intensity microneedling resulted in limited CaHA deposition (Supplementary Figure S5). When combined with enzymatic modulation, either by hyaluronidase injection (Supplementary Figure S6) or topical application before microneedling (Supplementary Figure S7), moderate improvements were observed. However, retention remained inferior to that achieved with intense protocols. The untreated negative control confirmed the absence of CaHA (Supplementary Figure S8), whereas the positive control, direct CaHA injection, showed dense and localized deposition within the dermis (Supplementary Figure S9). Collectively, these findings demonstrate that microneedling intensity is the dominant factor in determining intradermal CaHA distribution, with enzymatic pretreatment providing only modest enhancement under low-intensity conditions.

### 3.3. Fluorescence Imaging

**Figure 4:**
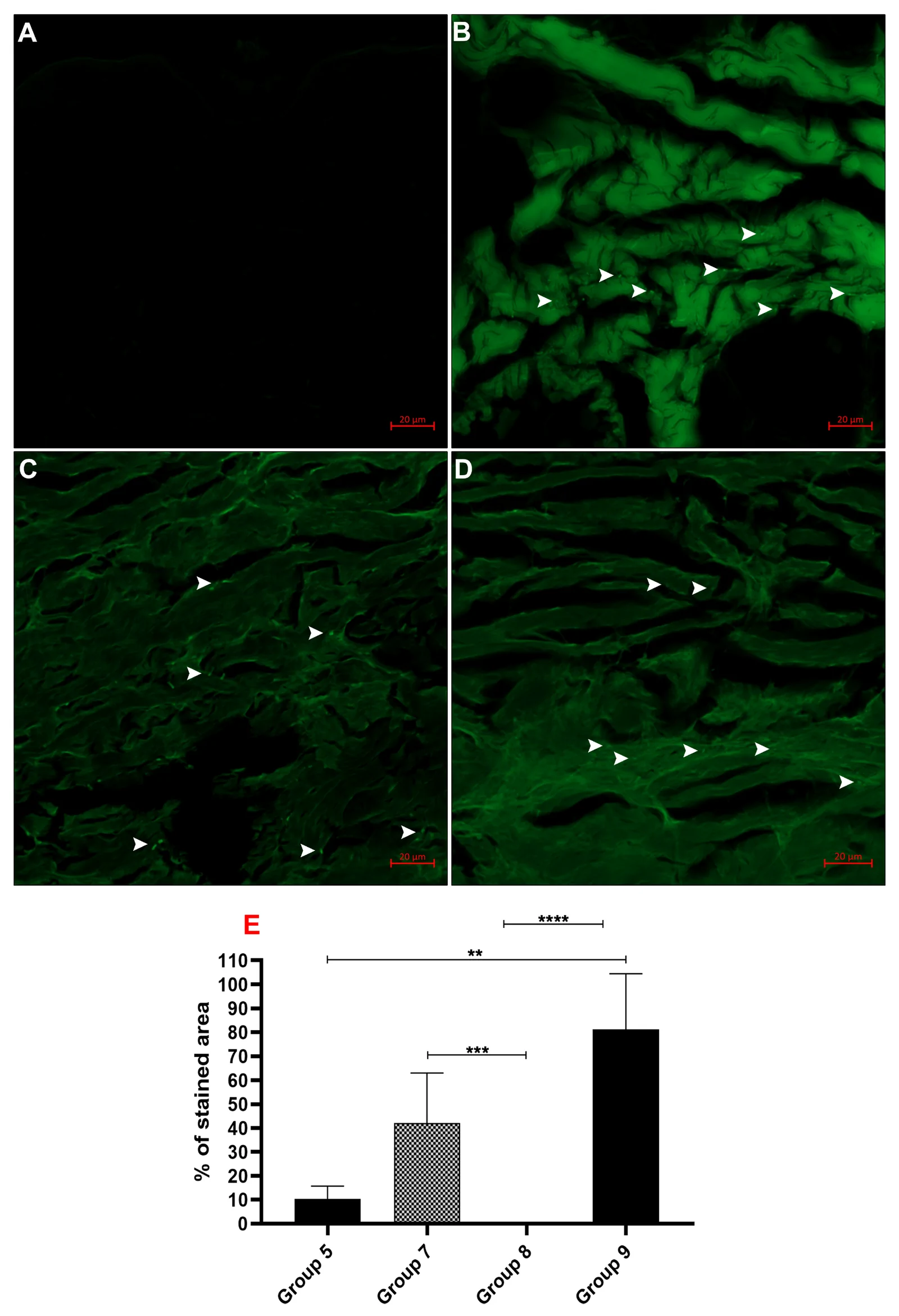
Confocal fluorescence microscopy of CaHA distribution was performed in porcine skin, with images acquired using a Zeiss LSM 980 microscope. An Alexa Fluor 488 laser with a wavelength of 517 nm was used. All images were taken using a 40× objective (1.2 μm) (scale bar = 20 μm). (**A**) Group 8—Negative control consisted of unprotected pig skin showing a dark background due to the absence of CaHA (40×). (**B**) Group 9—Positive control was carried out with a direct injection of 0.1 mL of CaHA without hyaluronidase (40×). (**C**) Group 5—Light microneedling (30 passes) was performed with a pen included with 0.1 mL of CaHA (40×). (**D**) Group 7—Light microneedling (30 passes) was performed with topical hyaluronidase followed by a pen with CaHA (40×). White arrowheads indicate CaHA particles. (**E**) Statistical analyses of CaHA particle distribution among groups were conducted using Graphpad PRISMA software version 8.0. Threshold-based ImageJ quantification (range 20–121 AU) was applied uniformly across samples. Negative control (G8) showed an absence of fluorescence, whereas direct injection (G9) revealed strong intradermal localization. Light microneedling alone (G5) showed minimal signal, while hyaluronidase pretreatment (G7) increased CaHA retention by approximately threefold compared to G5 (*p* < 0.001). Data represent the mean ± SD of three independent experiments. Data are shown as the mean ± SD of three independent experiments. ** *p* < 0.001; *** *p* < 0.0001; **** *p* < 0.00001.

Because conventional histology may fail to detect low levels of retained particles, confocal fluorescence microscopy was used to further assess CaHA distribution across the groups. As expected, the negative control showed no fluorescence (Supplementary Figure S10), whereas the positive control revealed strong localization of CaHA (Supplementary Figure S11). In groups treated with light microneedling, CaHA particles were scarcely detected when applied alone (Supplementary Figure S12). However, pretreatment with topical hyaluronidase markedly improved dermal retention, with fluorescence signals comparable to direct injection (Supplementary Figure S13). Quantitative analysis confirmed that enzymatic facilitation significantly enhanced intradermal CaHA distribution under low-intensity conditions (Figure 4).

### 3.4. Biological Activity of CaHA

**Figure 5:**
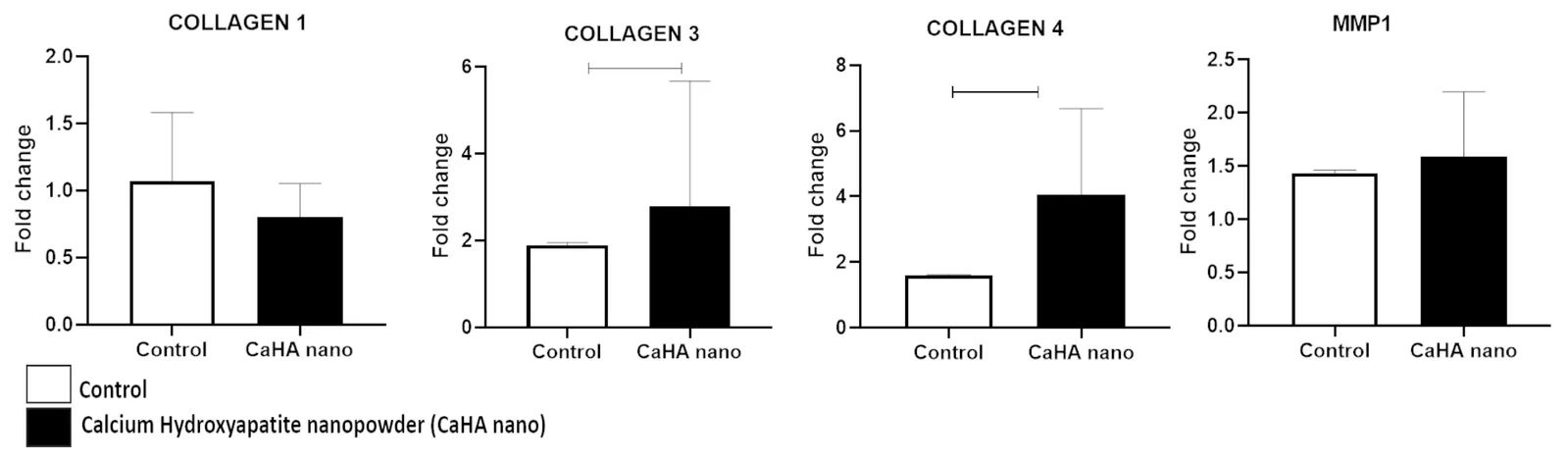
Analysis of gene expression of collagen types I, III, IV, and MMP-1 after CaHA treatment was performed on the QuantStudio 6 Flex RT-PCR equipment using a Taqman kit from Applied Biosystems by Thermo Fisher Scientific. Relative mRNA expression levels of collagen I, III, IV, and MMP-1 in HaCaT cells after exposure to CaHA nanoparticles are depicted. Group 8 (negative control) represents untreated cells, while the experimental groups were treated with CaHA. CaHA treatment significantly increased the expression of collagen III and IV compared to the control. COL3A1 expression increased ~2.3-fold and COL4A1 ~1.9-fold compared to the control, while COL1A1 and MMP1 remained unchanged. The results are expressed as the mean ± standard deviation of three independent experiments. Statistical significance is indicated by bars, with *p* < 0.05. Graphpad Prisma software version 8.0 was used for statistical analyses.

At the molecular level, HaCaT keratinocytes exposed to CaHA exhibited significant upregulation of COL3A1 and COL4A1 mRNA expression, both critical markers of extracellular matrix remodeling and dermal–epidermal junction integrity. No significant changes were observed in MMP1 or COL1A1, indicating a selective stimulation of collagens essential for dermal regeneration, rather than a generalized activation of matrix turnover (Figure 5).

## 4. Discussion

The findings of this study contribute to the growing body of evidence supporting microneedling as an effective method for transepidermal delivery of CaHA, enhancing its penetration into the skin and stimulating collagen synthesis. By combining microneedling with CaHA application, this approach introduces an innovative strategy in regenerative medicine. Unlike conventional rejuvenation techniques that rely on thermal energy or solely on injectable fillers, microneedling creates controlled microchannels that allow direct deposition of the biomaterial into the dermis [23]. This not only improves the bioavailability of CaHA but also selectively induces the expression of collagen types III and IV [24], which are critical for extracellular matrix remodeling and dermal–epidermal junction integrity [24]. Overall, the technique emerges as a clinically relevant, minimally invasive, and translational option for skin rejuvenation and repair in regenerative medicine. The experimental design was structured to compare microneedling intensities, hyaluronidase modulation, and delivery methods, since intense microneedling enhances penetration through deeper microchannels [25], hyaluronidase facilitates extracellular matrix permeability and particle diffusion [16], filler-loaded microneedling devices improve dermal deposition [26], and light microneedling offers a clinically relevant, less traumatic alternative [23]. At the same time, direct CaHA injection remains the gold standard for dermal delivery [14].

The ability of microneedling to create controlled microchannels in the epidermis and dermis facilitates the diffusion of bioactive compounds while simultaneously triggering an innate wound-healing response that leads to ECM remodeling [1,26]. This dual function highlights the potential of microneedling as a valuable tool in regenerative medicine and aesthetics [27]. The results demonstrate that microneedling alone is sufficient to enhance CaHA penetration, with high-intensity microneedling leading to significant CaHA deposition in the tissue, regardless of the presence of hyaluronidase. These findings suggest that mechanical disruption of the epidermal barrier is the primary determinant of CaHA delivery, aligning with previous studies highlighting microneedling’s role in facilitating the absorption of therapeutic agents [14]. However, while high-intensity microneedling enhances CaHA retention, histological analysis revealed considerable tissue damage in these groups, raising concerns about excessive mechanical disruption leading to adverse effects such as inflammation, fibrosis, or prolonged recovery time [28]. This underscores the need to establish an optimal microneedling protocol that maximizes efficacy while minimizing tissue trauma.

Microneedling-assisted delivery of CaHA effectively stimulates collagen production by introducing biostimulatory fillers into the dermis, promoting neocollagenesis and tissue remodeling [26]. Similarly, combining microneedling with platelet-rich plasma (PRP) leverages growth factors to enhance collagen synthesis and accelerate healing. Each approach offers unique advantages and limitations, necessitating consideration of individual patient needs and treatment goals [29]. Radiofrequency (RF) treatments utilize electromagnetic waves to generate dermal heat, inducing controlled thermal injury and subsequent collagen remodeling [30]. While effective in stimulating collagen types I and III, RF does not directly deliver bioactive compounds like CaHA, limiting targeted regenerative responses [30].

Additionally, RF outcomes may vary due to individual differences in skin thickness and hydration levels [30]. Interestingly, Photobiomodulation therapies, particularly fractional laser resurfacing, create controlled microthermal zones that trigger wound healing, effectively reducing fine lines, wrinkles, and scars [31]. However, they are associated with significant downtime, risk of post-inflammatory hyperpigmentation, and limited suitability for darker skin tones. In contrast, microneedling causes less epidermal damage and allows concurrent application of regenerative substances like CaHA, offering a customizable approach to delivering biostimulatory agents [31]. PRP therapy involves applying autologous growth factors to stimulate fibroblast activity and increase extracellular matrix components, including collagen types I and III [32]. However, PRP efficacy can be variable due to differences in platelet concentrations, preparation methods, and patient-specific factors. Unlike CaHA, PRP does not provide structural dermal support, and its effects are often transient, requiring multiple sessions for sustained results [32]. Injectable biostimulatory fillers like poly-L-lactic acid (PLLA) induce neocollagenesis through a controlled inflammatory response, acting as scaffolds that stimulate fibroblast proliferation and collagen deposition over several months [33]. Unlike traditional injection methods, which require subdermal placement to avoid dermal irregularities, microneedling creates controlled microchannels that directly diffuse CaHA particles into the dermal layer [10].

The comparison between microneedling-assisted CaHA delivery and these alternative techniques highlights its unique advantages [26]. Unlike RF and laser therapies, which rely on thermal energy to induce collagen remodeling, microneedling enables direct mechanical penetration of CaHA into the dermis, ensuring precise deposition at targeted depths [34]. Compared to PRP [35], CaHA offers a more consistent and predictable regenerative response by providing an exogenous biostimulatory agent that actively induces collagen synthesis [4]. Furthermore, unlike PLLA, which requires gradual resorption and macrophage-mediated neocollagenesis following the delayed breakdown of its microspheres [36,37], CaHA integrates immediately into soft tissues and promotes sustained extracellular matrix remodeling, with increased collagen deposition observed as early as 30 days and maintained up to 90 days post-injection [38]. These characteristics suggest that microneedling-assisted CaHA delivery may offer an optimal balance of efficacy, safety, and versatility for skin rejuvenation and tissue regeneration.

Despite these advantages, several limitations must be considered. The study was conducted on ex vivo skin models and immortalized keratinocyte cell lines, which may not fully replicate the complexity of in vivo biological responses. Future clinical studies must validate these findings and assess the long-term efficacy and safety of microneedling-mediated CaHA delivery in human subjects. Another important consideration is the potential for combining CaHA with other regenerative agents, such as growth factors, peptides, or PRP, to enhance its biostimulatory effects [10,39]. Synergistic approaches that leverage multiple regenerative pathways could provide superior clinical outcomes and expand the applicability of microneedling-assisted CaHA delivery beyond aesthetic medicine into wound healing, scar revision, and tissue regeneration [39].

Additionally, further investigations should focus on elucidating the molecular mechanisms underlying CaHA-induced collagen synthesis to refine treatment strategies and improve predictability in clinical practice. This study’s results suggest that topical hyaluronidase under low-intensity microneedling enhances the distribution and retention of CaHA within the ECM, potentially compensating for reduced mechanical penetration. This aligns with previous findings indicating that hyaluronidase can modulate ECM permeability and facilitate the diffusion of macromolecules by transiently degrading hyaluronic acid-rich barriers [39]. A limitation of this study is the lack of in vivo or clinical validation. Although the present findings provide essential in vitro insights, future studies should include animal models and clinical investigations to validate the translational applicability of the results.

On average, microneedling procedures can reach depths of up to 4 mm. However, in the case of high-intensity microneedling, the main factor is not only the depth of penetration but also the degree of tissue disruption. In the present ex vivo study, tissue disruption was assessed qualitatively, which represents a methodological limitation, as inflammatory or molecular markers could not be evaluated. From a clinical perspective, this distinction is crucial: while controlled microinjury is necessary to stimulate collagen production, excessive tissue damage may increase the risk of fibrosis or scarring. Therefore, establishing the delicate balance between efficacy and safety remains challenging in this post-mortem model, underscoring the translational need for in vivo validation.

In conclusion, this study demonstrates that microneedling effectively enhances the transepidermal delivery of CaHA, promoting the expression of collagen types III and IV while preserving ECM integrity. High-intensity microneedling increases CaHA penetration but may lead to tissue damage, emphasizing the need for optimized treatment protocols. The role of hyaluronidase appears limited under these conditions, but it may enhance CaHA retention and distribution when mechanical penetration is reduced. Microneedling-assisted CaHA delivery offers a unique combination of precise mechanical penetration and direct biostimulation in the dermis with minimal invasiveness compared to other collagen stimulation techniques, such as RF and laser treatment. These findings support its clinical potential for skin rejuvenation and regenerative medicine. Further research is necessary to refine treatment parameters, confirm long-term safety, and explore combination therapies for improved regenerative outcomes.

## Data Availability

The original contributions presented in this study are included in the article/Supplementary Material. Further inquiries can be directed to the corresponding author.

## Supplementary Materials

The following supporting information can be downloaded at: https://www.mdpi.com/article/10.3390/biomedicines13102463/s1, Figure S1: Topical application of 0.1 mL CaHA followed by intense microneedling for 1 min, without hyaluronidase. Images at (A) 10× and (B) 40× show initial CaHA deposition with limited tissue integration; Figure S2: Topical application of 0.1 mL CaHA followed by intense microneedling for 1 min, with topical hyaluronidase. Images at (A) 10× and (B) 40× reveal improved CaHA dispersion and dermal distribu-tion compared with Supplementary Figure S1; Figure S3: CaHA (0.1 mL) aspirated into the microneedling pen and applied with intense mi-croneedling for 1 min, without hyaluronidase. Images at (A) 10× and (B) 40× demonstrate more homogeneous delivery than topical application; Figure S4: CaHA (0.1 mL) aspirated into the microneedling pen and applied with intense mi-croneedling for 1 min, with topical hyaluronidase. Images at (A) 10× and (B) 40× show enhanced penetration and greater dermal retention compared with Supplementary Figure S3; Figure S5: Light microneedling (30 repetitions) using a pen loaded with 0.1 mL CaHA. Images at (A) 10× and (B) 40× indicate minimal CaHA deposition and weak tissue response; Figure S6: Injection of 0.1 mL hyaluronidase, followed by light microneedling (30 repetitions) with a pen loaded with 0.1 mL CaHA. Images at (A) 10× and (B) 40× reveal moderate CaHA dispersion, similar to topical hyaluronidase; Figure S7: Topical hyaluronidase applied before light microneedling (30 repetitions) with a pen loaded with 0.1 mL CaHA. Images at (A) 10× and (B) 40× show significantly improved CaHA retention compared with Supplementary Figure S5; Figure S8: Negative control, untreated tissue. Images at (A) 10× and (B) 40× confirm absence of CaHA, with preserved tissue architecture; Figure S9: Positive control, direct injection of 0.1 mL CaHA without hyaluronidase. Images at (A) 10× and (B) 40× demonstrate dense CaHA deposition and strong dermal localization; Figure S10: Negative control, untreated ex vivo pig skin tissue. Images were obtained using a Zeiss LSM 980 Confocal Fluorescence Microscope. Figures (A) and (B) were taken using the Wide Field technique, while (C) and (D) were taken using the LSM Confocal technique. (A) 5× objective; (B) 20× objective; (C) 10× objective; (D) 40× objective; Figure S11: Positive control, direct injection of 0.1 mL CaHA without hyaluronidase. Images were obtained using a Zeiss LSM 980 Confocal Fluorescence Microscope. Figures (A) and (B) were Wide Field; (C) and (D) LSM Confocal. (A) 5× objective; (B) 20× objective; (C) 10× objective; (D) 40× objective. White arrowheads highlight CaHA particles; Figure S12: Light microneedling (30 repetitions) using a pen loaded with 0.1 mL CaHA. The pro-cedure was conducted in a homogeneous manner to promote material distribution. Images were obtained using a Zeiss LSM 980 Confocal Fluorescence Microscope. (A) 5× objective; (B) 20× objective; (C) 10× objective; (D) 40× ob-jective. White arrowheads highlight CaHA particles; Figure S13: Light microneedling (30 repetitions) with a pen, preceded by topical application of 0.1 mL hyaluronidase, followed by microneedling with a pen loaded with 0.1 mL CaHA. Images were obtained using a Zeiss LSM 980 Confocal Fluorescence Microscope. (A) 5× objective; (B) 20× objective; (C) 10× objective; (D) 40× ob-jective. White arrowheads highlight CaHA particles; Figure S14: Scanning Electron Microscopy (SEM) images of polyvinylpyrrolidone (PVP), acquired using a Thermo Fisher Prisma E microscope in High Vacuum mode. (A) PVP at 60× magnification (scale bar: 500 μm); (B) PVP at 375× magnification (scale bar: 500 μm).
