# Supplementary material for: Transepidermal Delivery of Calcium Hydroxyapatite (CaHA) Microneedling: A Novel Approach for Inducing Collagen Types III and IV
Source: Biomedicines. 2025 Oct 10;13(10):2463. doi: 10.3390/biomedicines13102463 (PMC12562168; doi:10.3390/biomedicines13102463)
Supplement: Supplementary file 1 [file biomedicines-13-02463-s001.zip › biomedicines-3737684-supplementary.pdf]

Supplementary Figure S1. Topical application of 0.1 mL CaHA followed by intense microneedling for 1 minute, without hyaluronidase. Images at (A) 10× and (B) 40× show initial CaHA deposition with limited tissue integration.

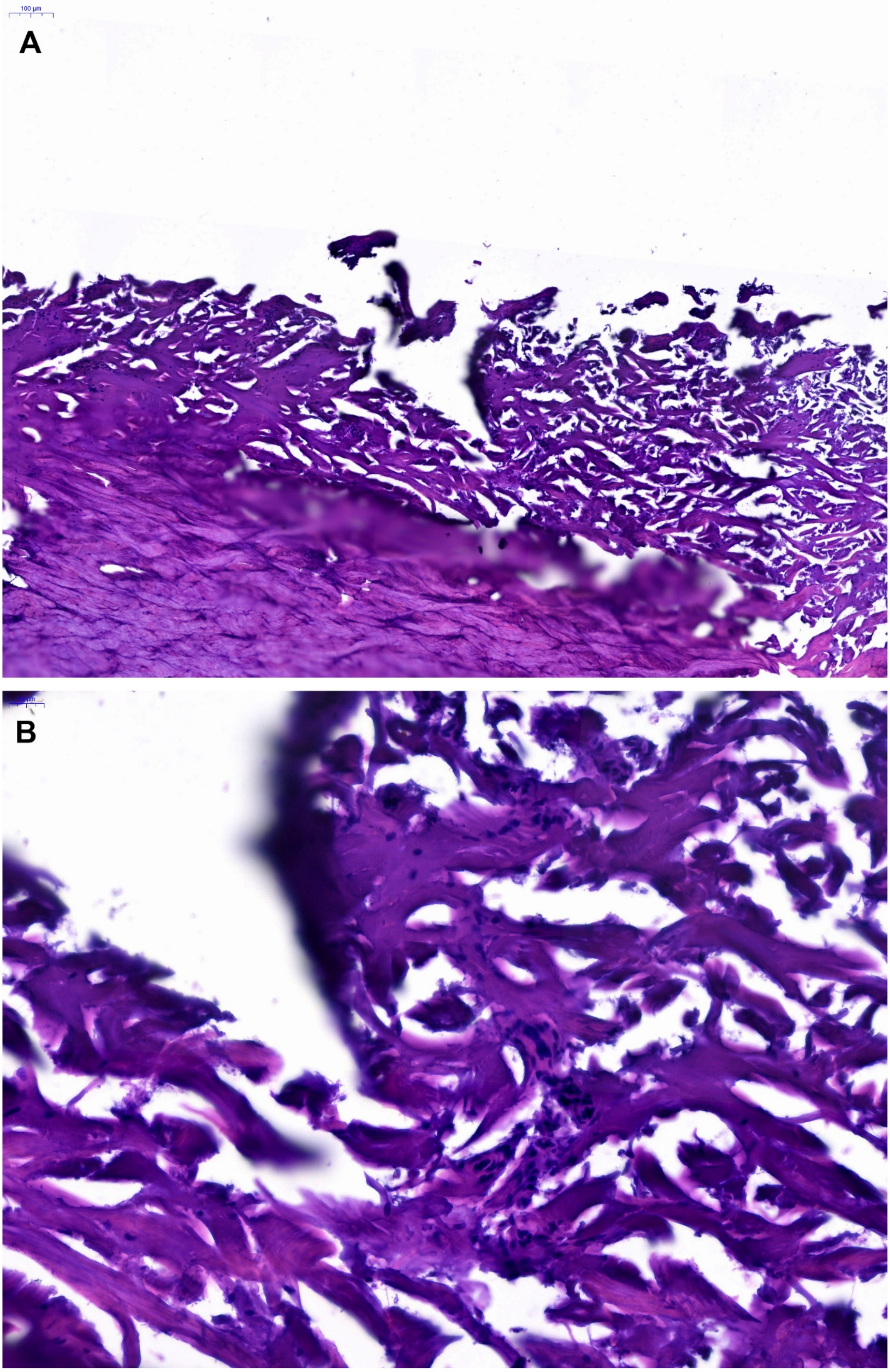

Supplementary Figure S2. Topical application of 0.1 mL CaHA followed by intense microneedling for 1 minute, with topical hyaluronidase. Images at (A) 10× and (B) 40× reveal improved CaHA dispersion and dermal distribution compared with Supplementary Figure S1.

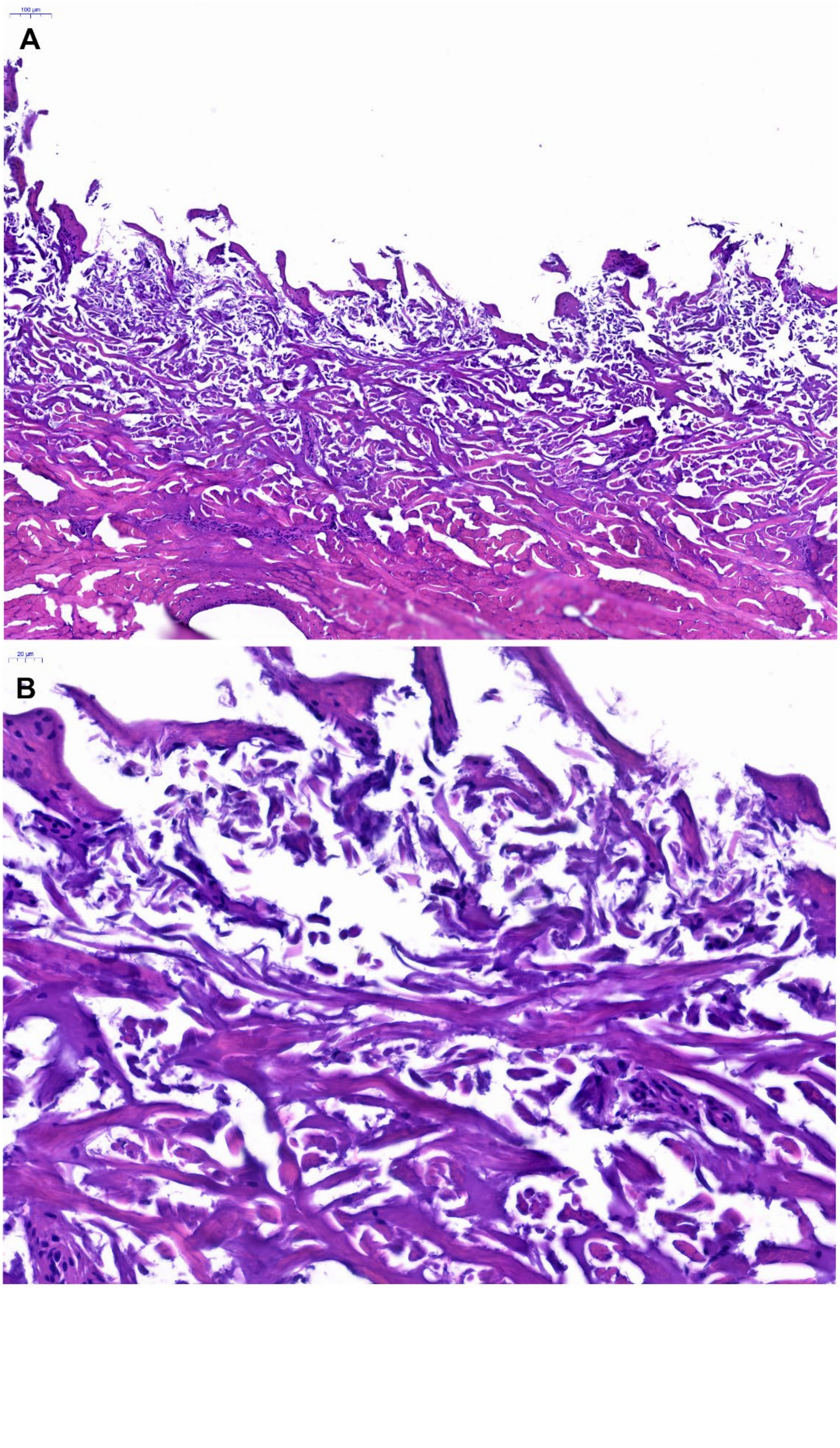

Supplementary Figure S3. CaHA (0.1 mL) aspirated into the microneedling pen and applied with intense microneedling for 1 minute, without hyaluronidase. Images at (A) 10× and (B) 40× demonstrate more homogeneous delivery than topical application.

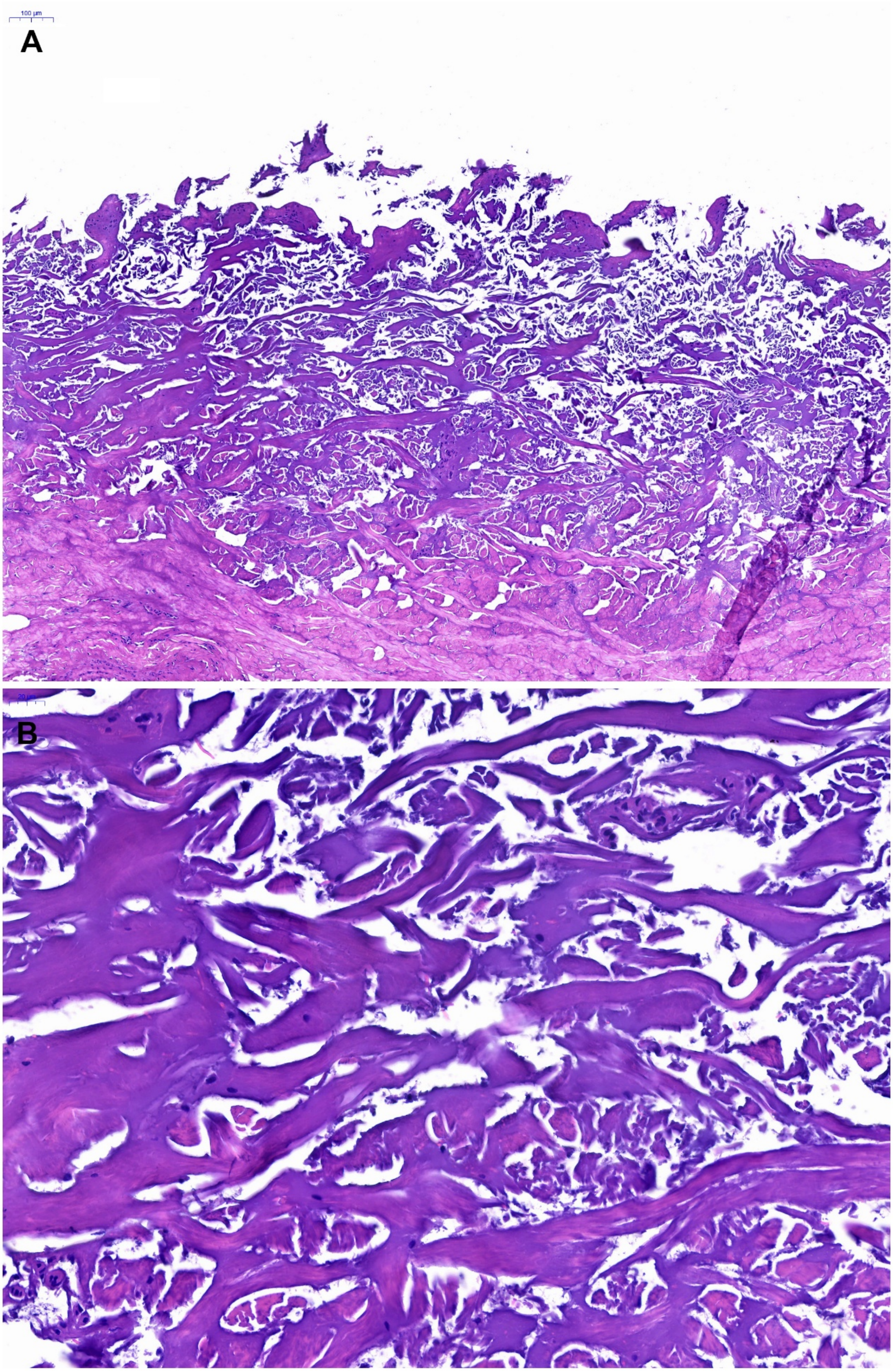

Supplementary Figure S4. CaHA (0.1 mL) aspirated into the microneedling pen and applied with intense microneedling for 1 minute, with topical hyaluronidase. Images at (A) 10× and (B) 40× show enhanced penetration and greater dermal retention compared with Supplementary Figure S3.

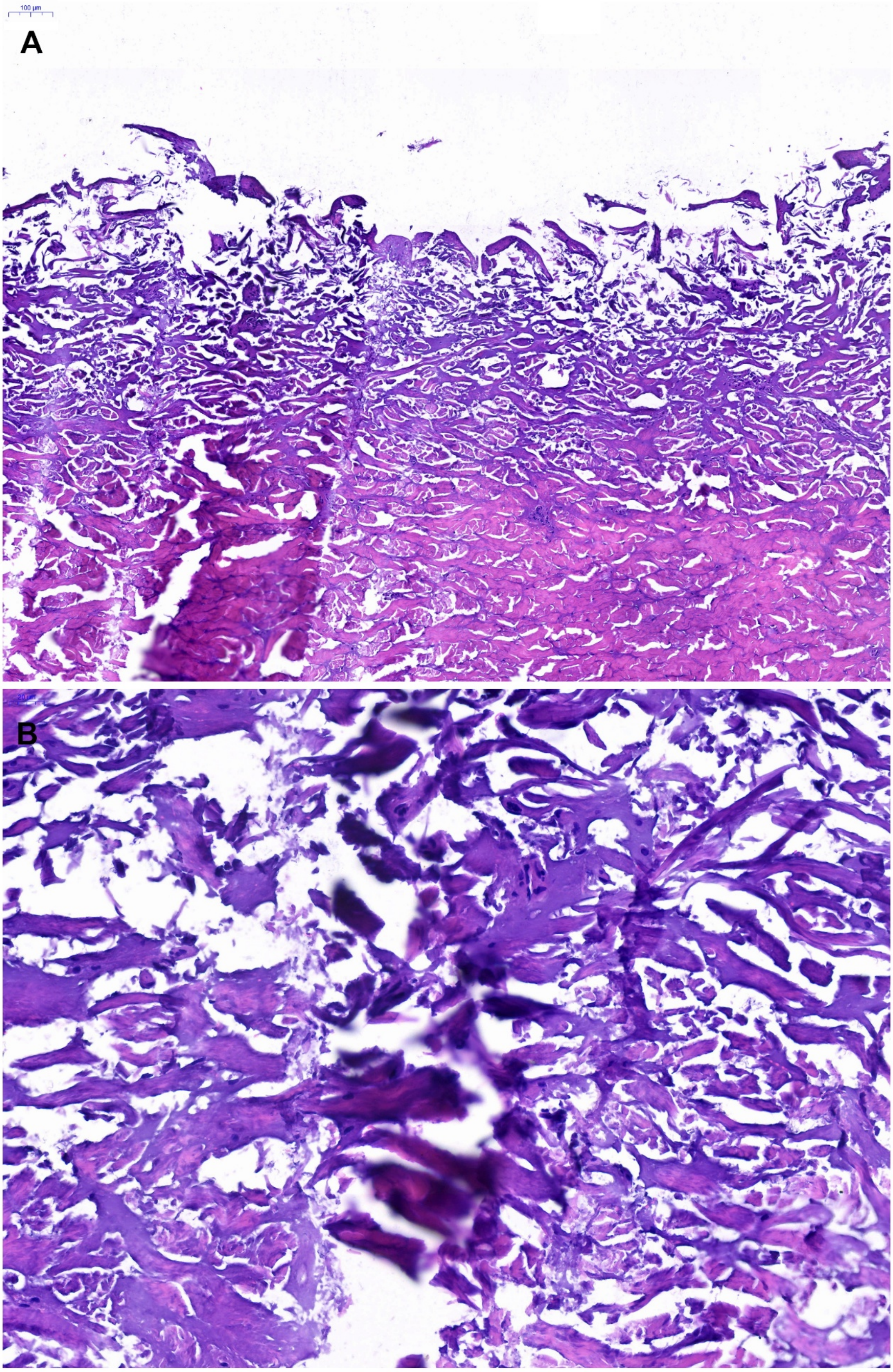

Supplementary Figure S5. Light microneedling (30 repetitions) using a pen loaded with 0.1 mL CaHA. Images at (A) 10× and (B) 40× indicate minimal CaHA deposition and weak tissue response.

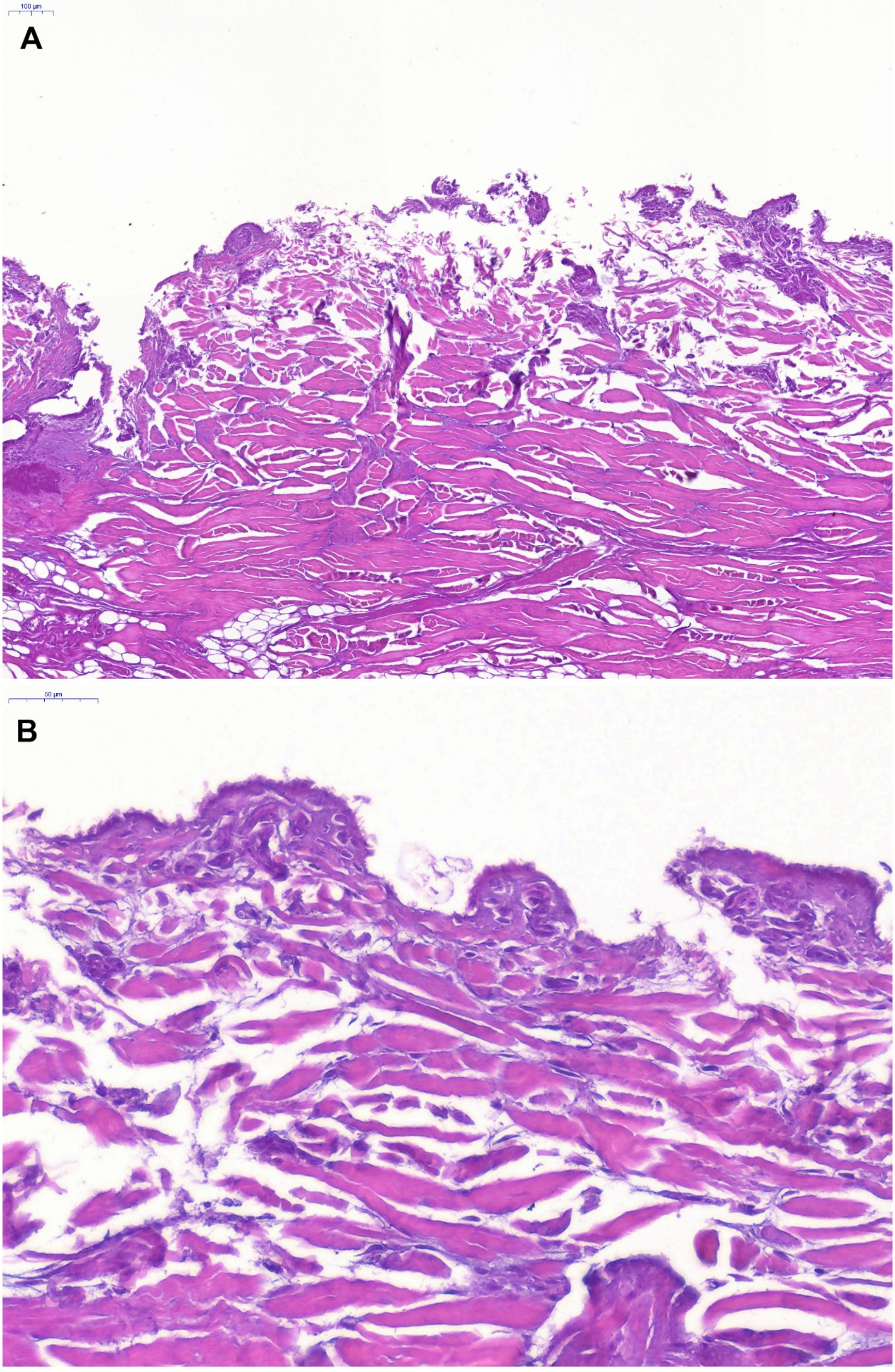

Supplementary Figure S6. Injection of 0.1 mL hyaluronidase, followed by light microneedling (30 repetitions) with a pen loaded with 0.1 mL CaHA. Images at (A) 10× and (B) 40× reveal moderate CaHA dispersion, similar to topical hyaluronidase.

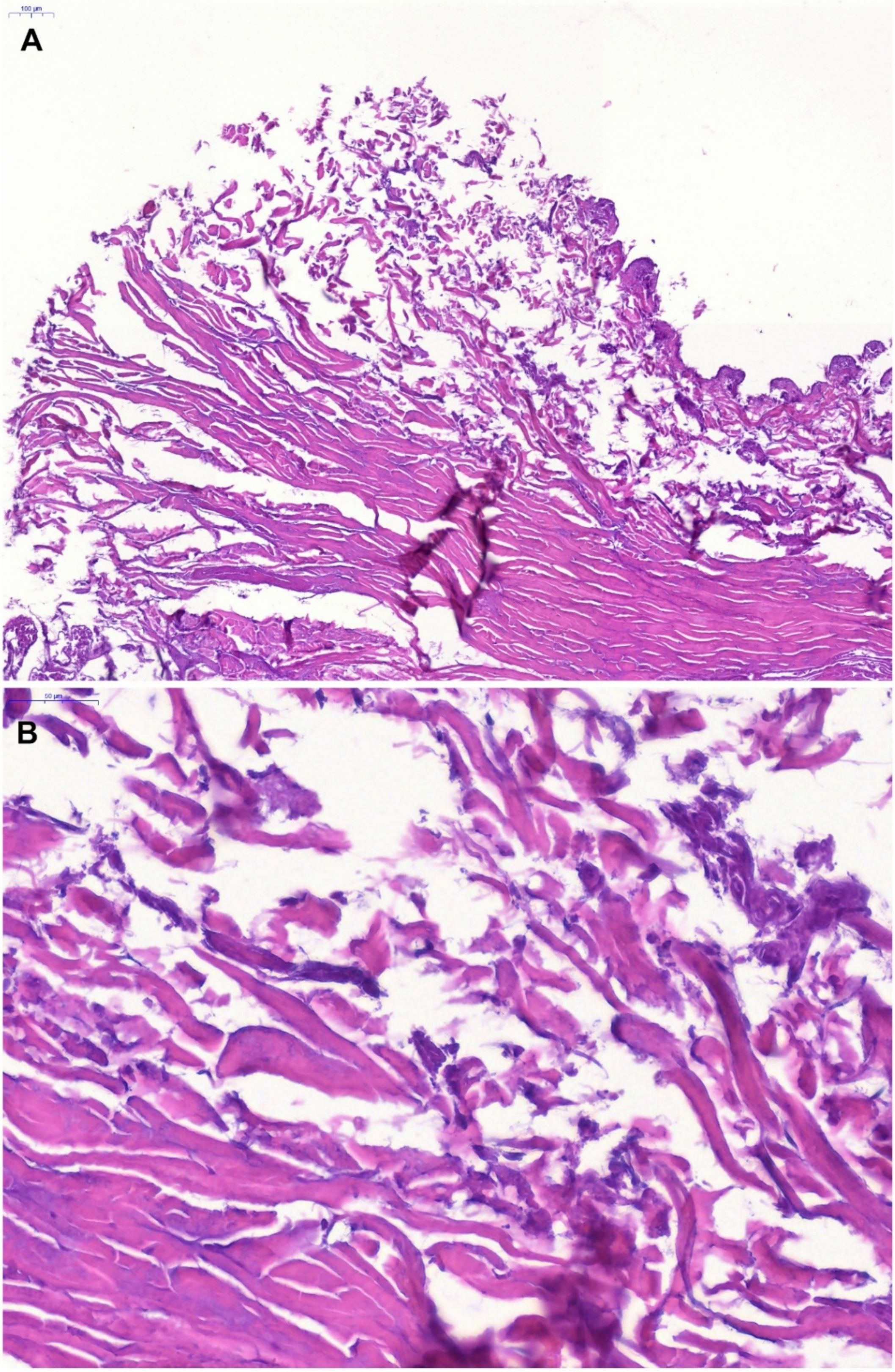

Supplementary Figure S7. Topical hyaluronidase applied before light microneedling (30 repetitions) with a pen loaded with 0.1 mL CaHA. Images at (A) 10× and (B) 40× show significantly improved CaHA retention compared with Supplementary Figure S5.

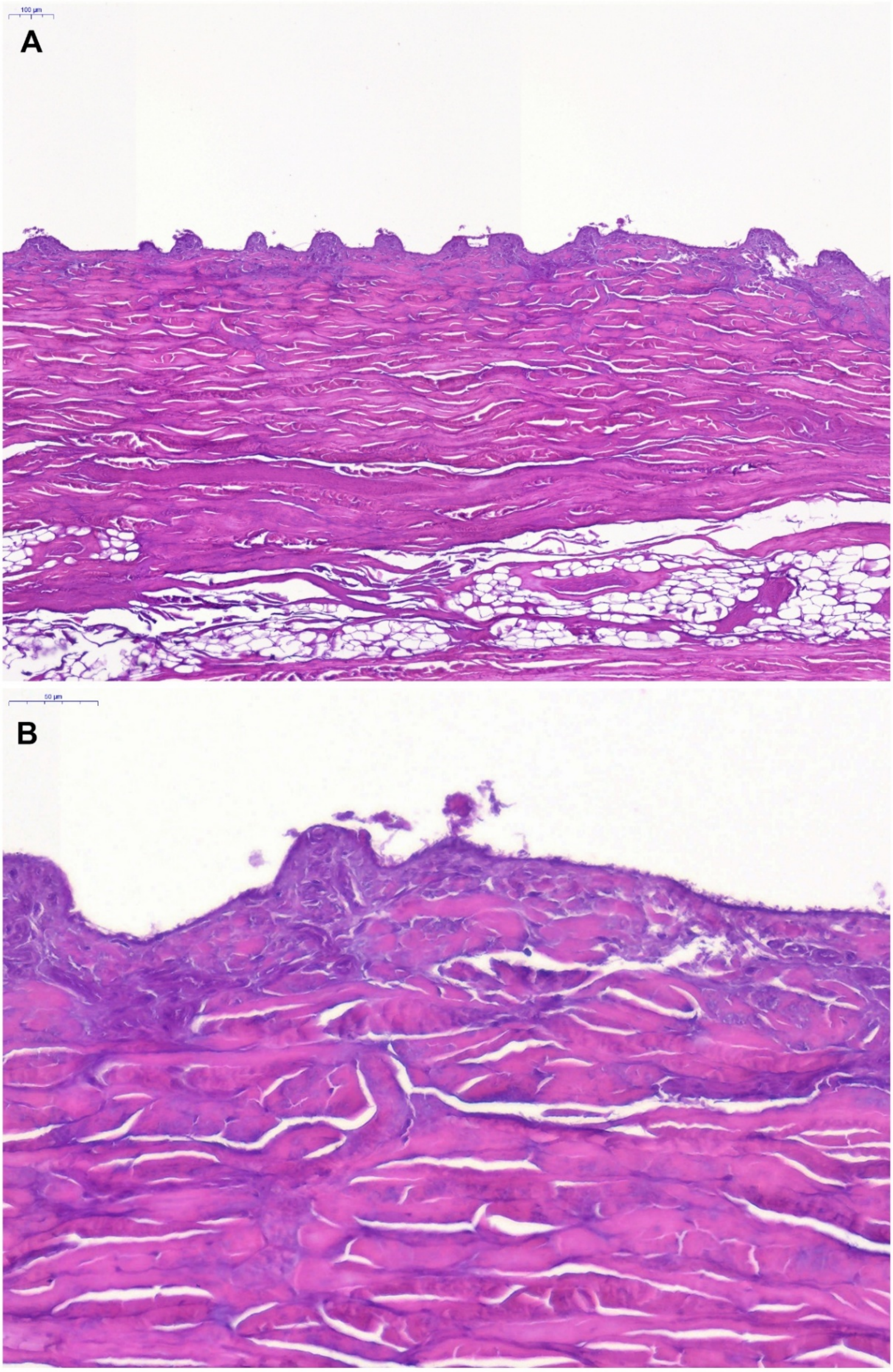

Supplementary Figure S8. Negative control, untreated tissue. Images at (A) 10× and (B) 40× confirm absence of CaHA, with preserved tissue architecture.

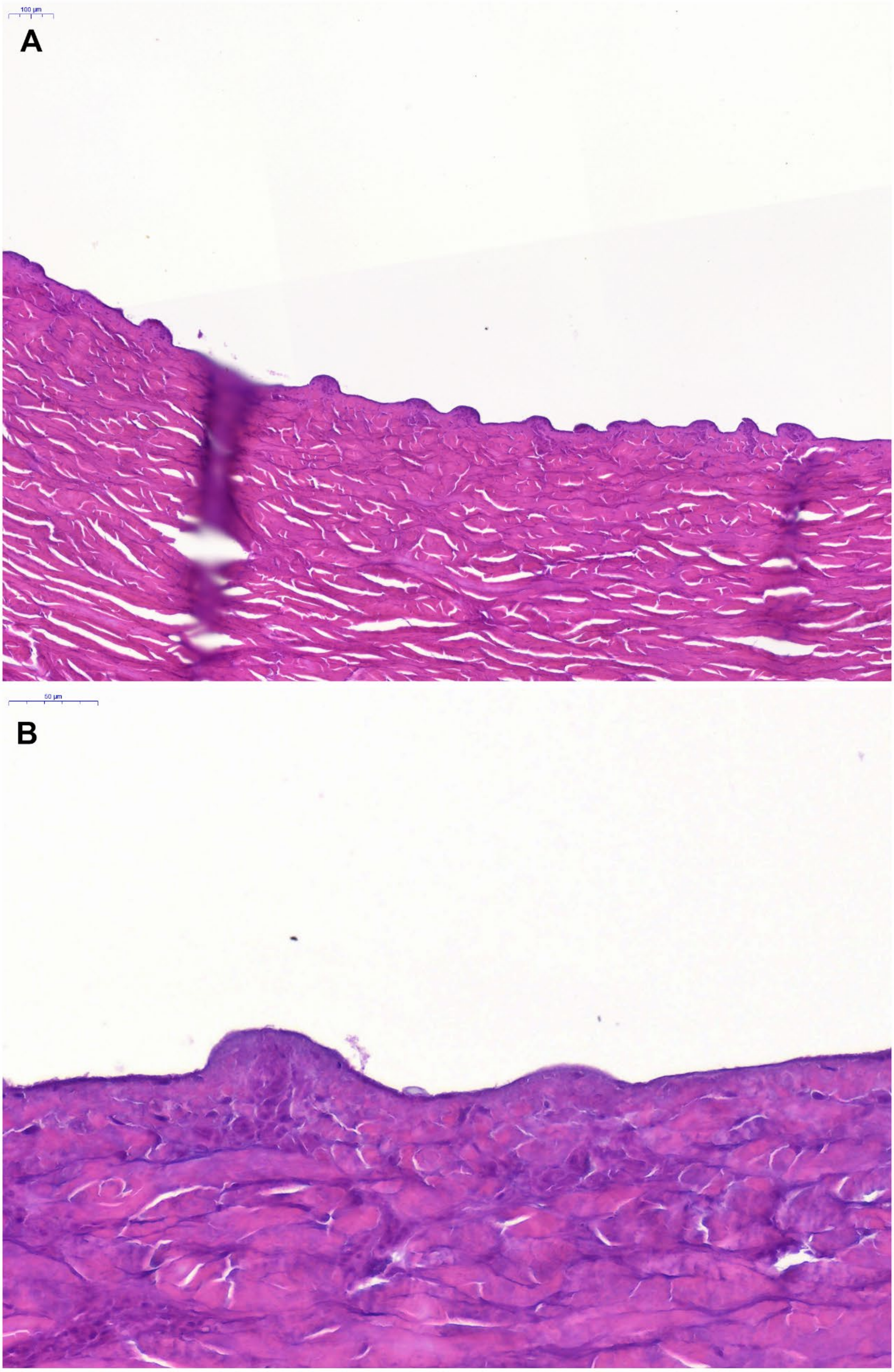

Supplementary Figure S9. Positive control, direct injection of 0.1 mL CaHA without hyaluronidase. Images at (A) 10× and (B) 40× demonstrate dense CaHA deposition and strong dermal localization.

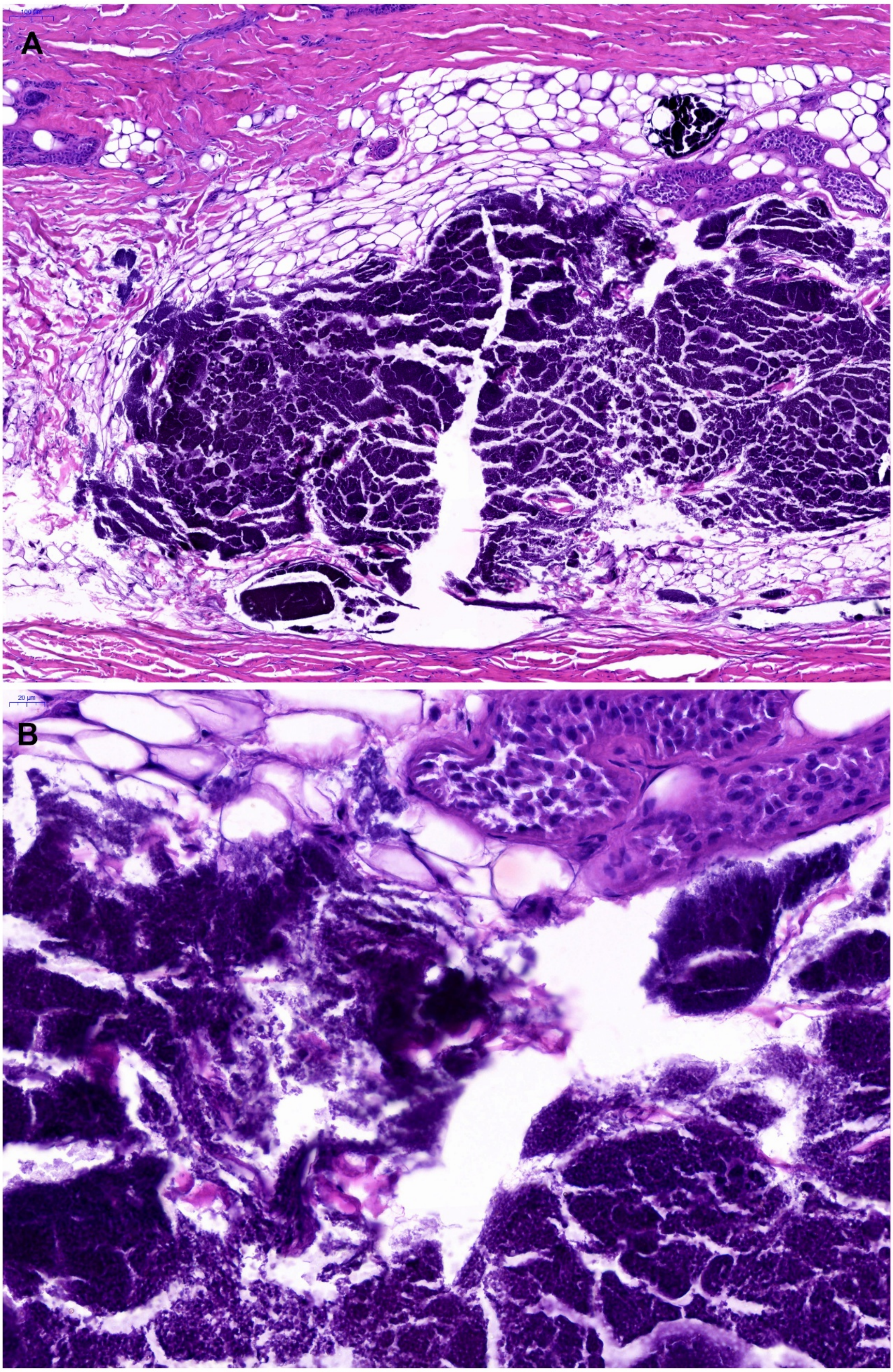

Supplementary Figure S10. Negative control, untreated ex vivo pig skin tissue. Images were obtained using a Zeiss LSM 980 Confocal Fluorescence Microscope. Figures (A) and (B) were taken using the Wide Field technique, while (C) and (D) were taken using the LSM Confocal technique. (A) Image obtained with the 5× objective; (B) Image obtained with the 20× objective; (C) Image obtained with the 10× objective; (D) Image obtained with the 40× objective.

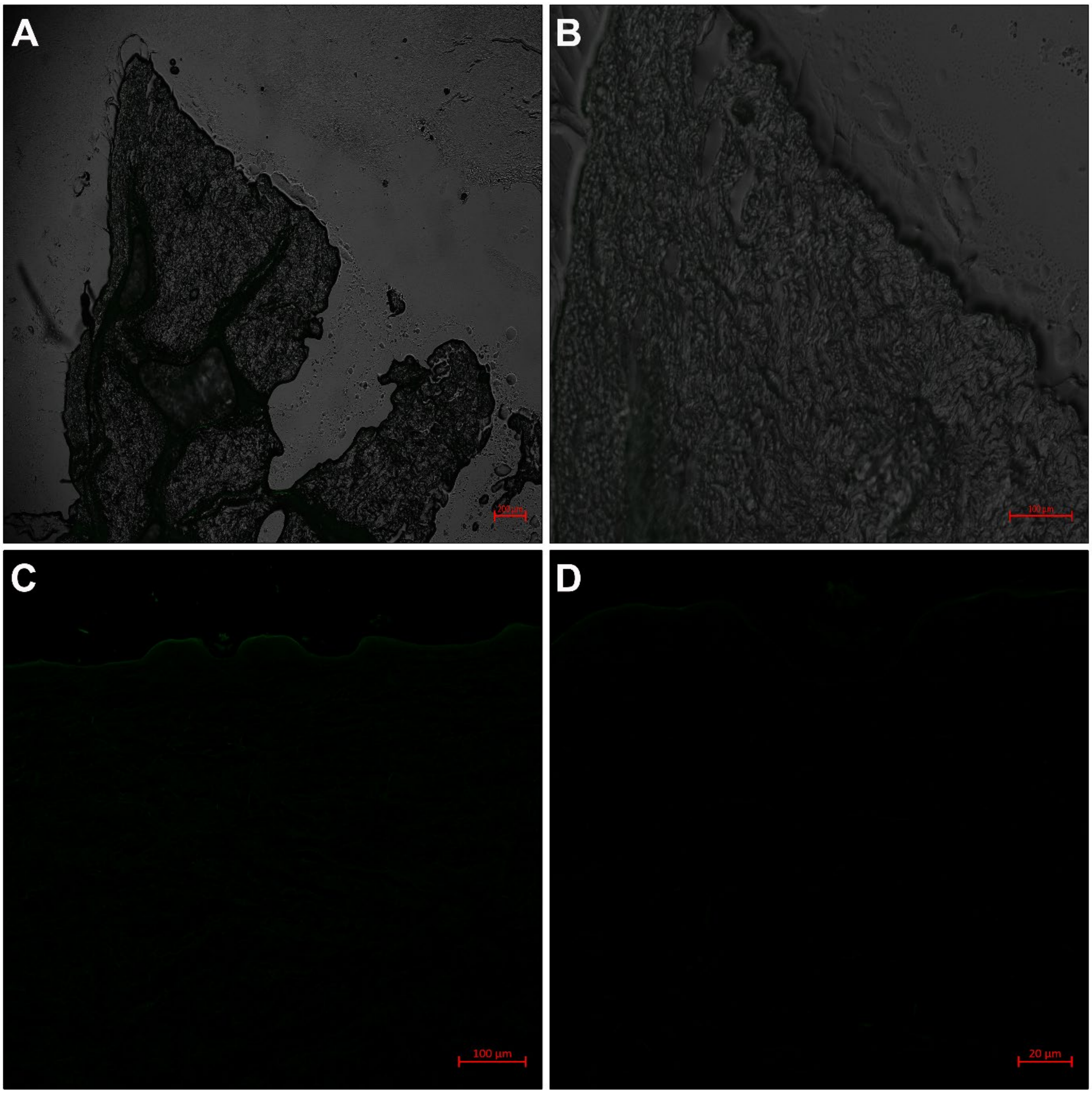

Supplementary Figure S11. Positive control, direct injection of 0.1 mL CaHA without hyaluronidase. Images were obtained using a Zeiss LSM 980 Confocal Fluorescence Microscope. Figures (A) and (B) were taken using the Wide Field technique, while (C) and (D) were taken using the LSM Confocal technique. (A) Image obtained with the 5× objective; (B) Image obtained with the 20× objective; (C) Image obtained with the 10× objective; (D) Image obtained with the 40× objective. The white arrowheads highlight CaHA particles.

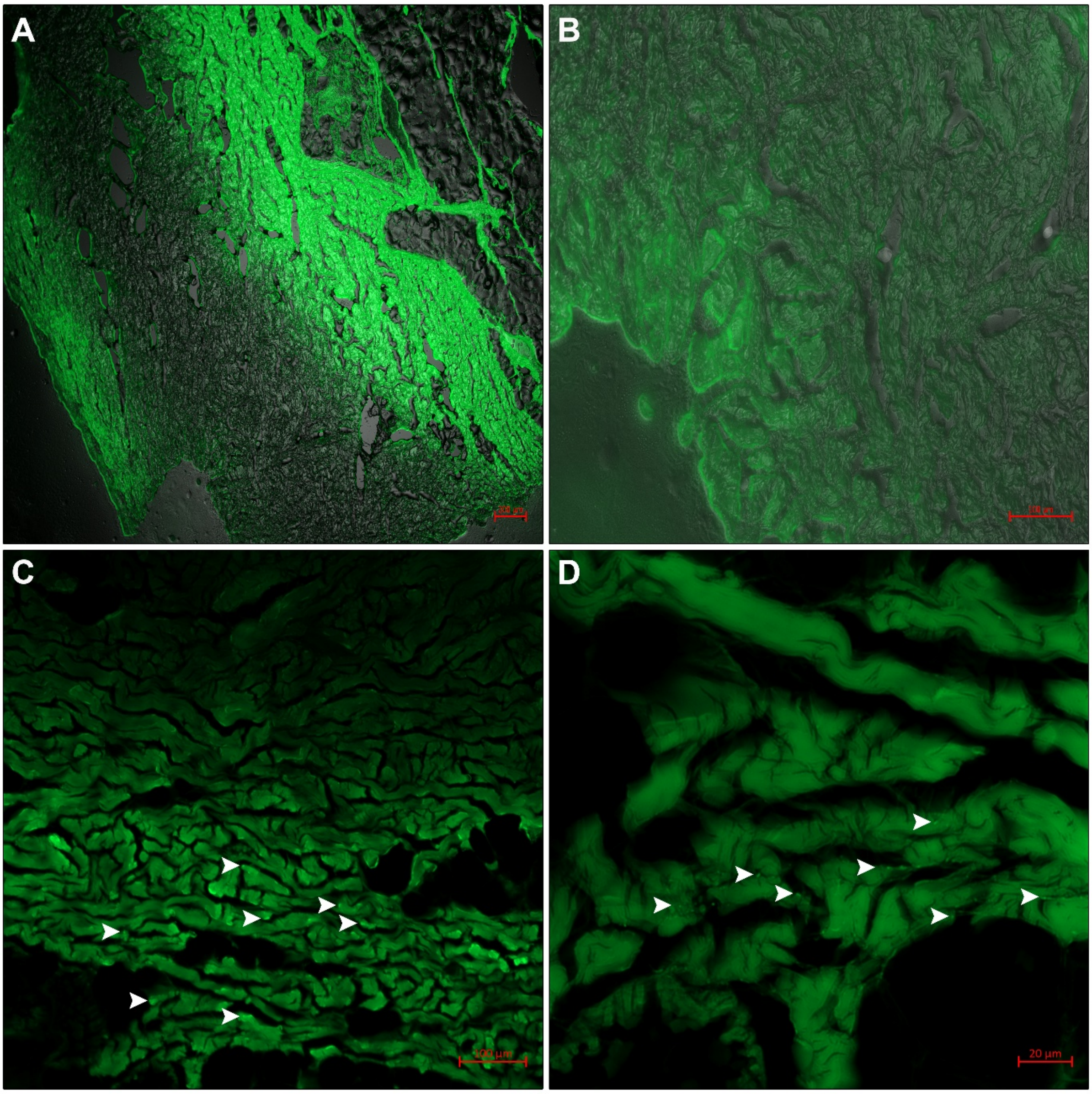

Supplementary Figure S12. Light microneedling (30 repetitions) using a pen loaded with 0.1 mL CaHA. The procedure was conducted in a homogeneous manner to promote material distribution. Images were obtained using a Zeiss LSM 980 Confocal Fluorescence Microscope. Figures (A) and (B) were taken using the Wide Field technique, while (C) and (D) were taken using the LSM Confocal technique. (A) Image obtained with the 5× objective; (B) Image obtained with the 20× objective; (C) Image obtained with the 10× objective; (D) Image obtained with the 40× objective. The white arrowheads highlight CaHA particles.

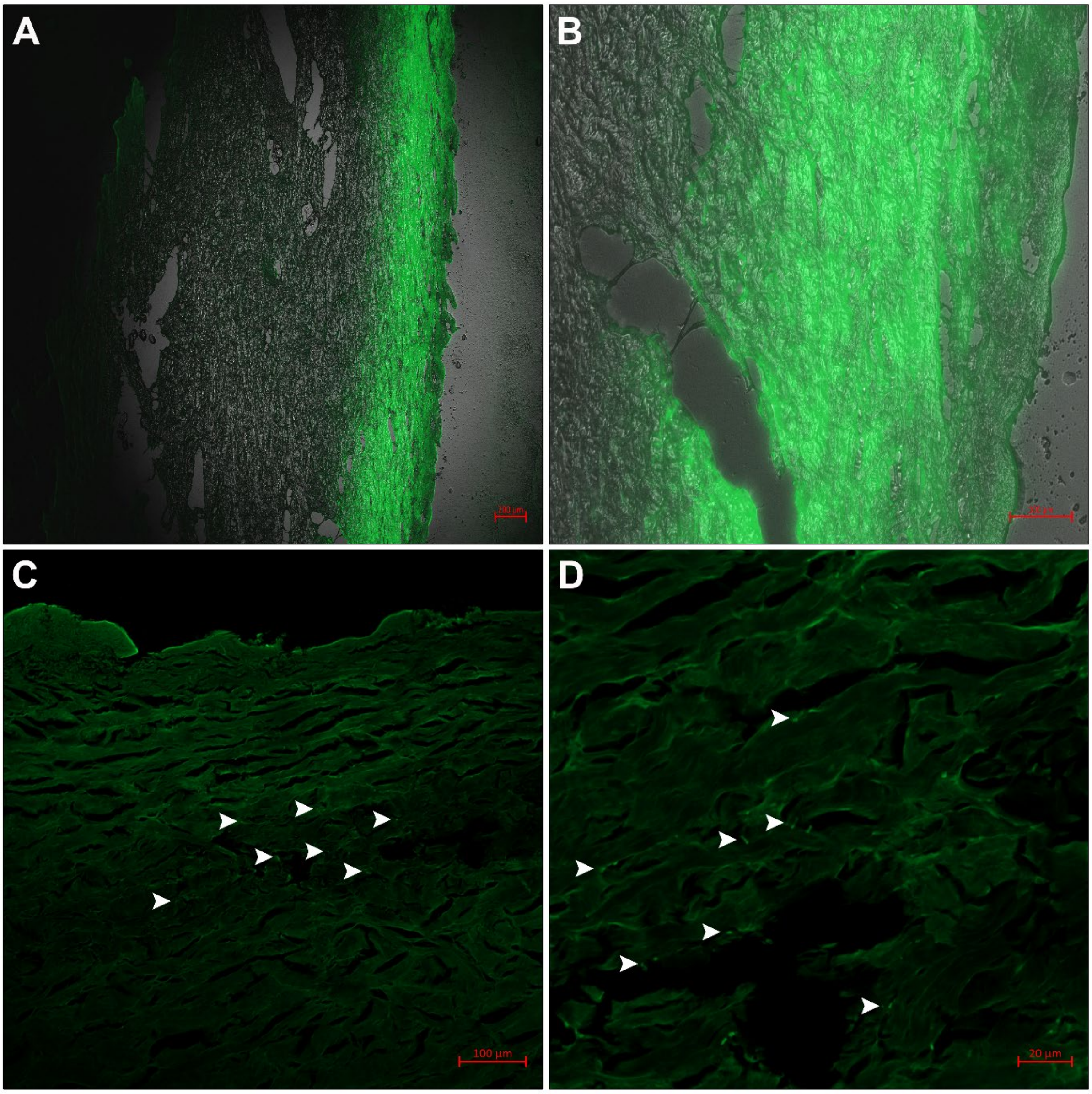

Supplementary Figure S13. Light microneedling (30 repetitions) with a pen, preceded by topical application of 0.1 mL hyaluronidase, followed by microneedling with a pen loaded with 0.1 mL CaHA. Images were obtained using a Zeiss LSM 980 Confocal Fluorescence Microscope. Figures (A) and (B) were taken using the Wide Field technique, while (C) and (D) were taken using the LSM Confocal technique. (A) Image obtained with the 5× objective; (B) Image obtained with the 20× objective; (C) Image obtained with the 10× objective; (D) Image obtained with the 40× objective. The white arrowheads highlight CaHA particles.

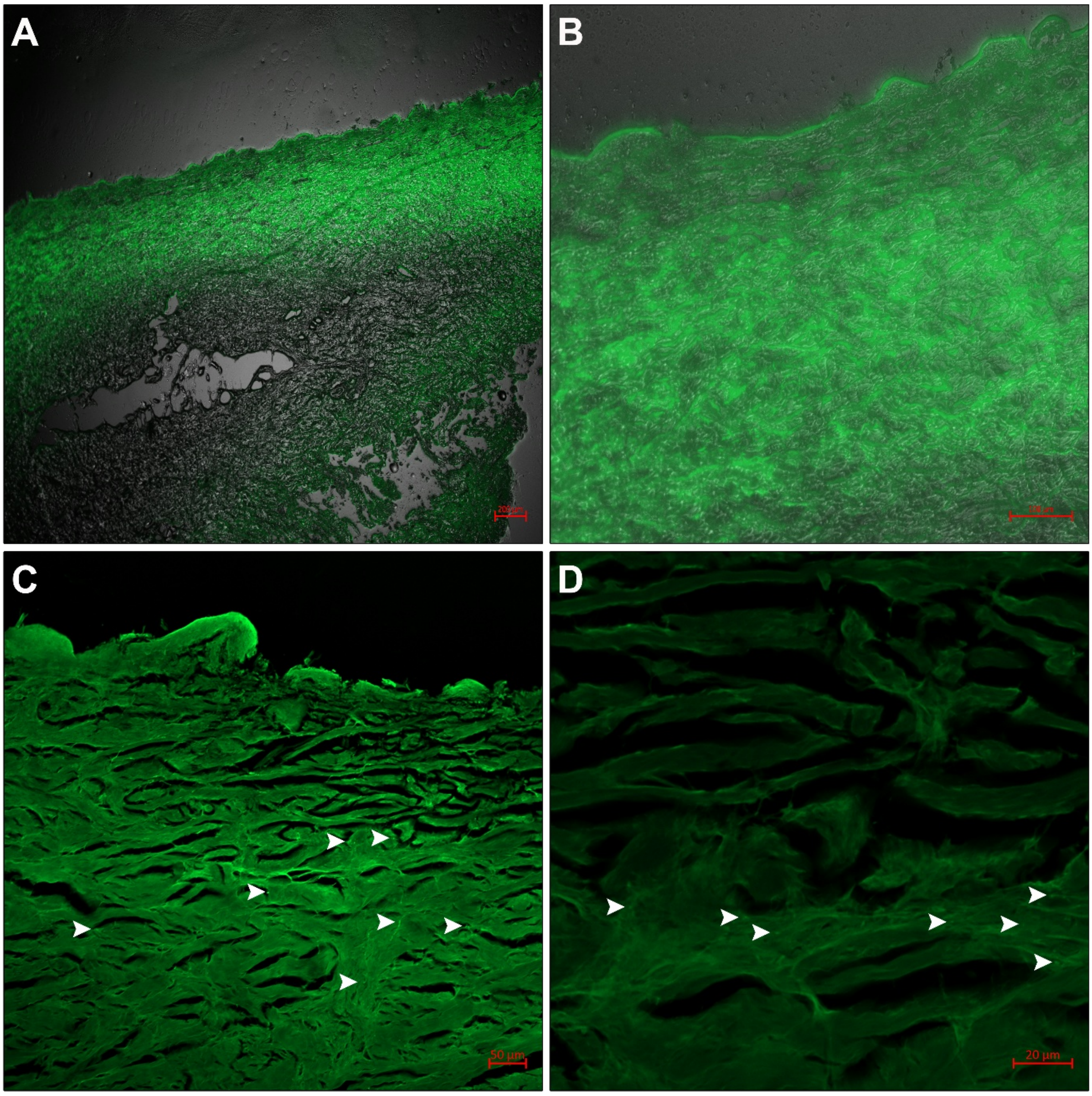

Supplementary Figure S14. Scanning Electron Microscopy (SEM) images of polyvinylpyrrolidone (PVP), acquired using a Thermo Fisher Prisma E microscope in High Vacuum mode. (A) PVP at 60× magnification (scale bar: 500 µm); (B) PVP at 375× magnification (scale bar: 500 µm).

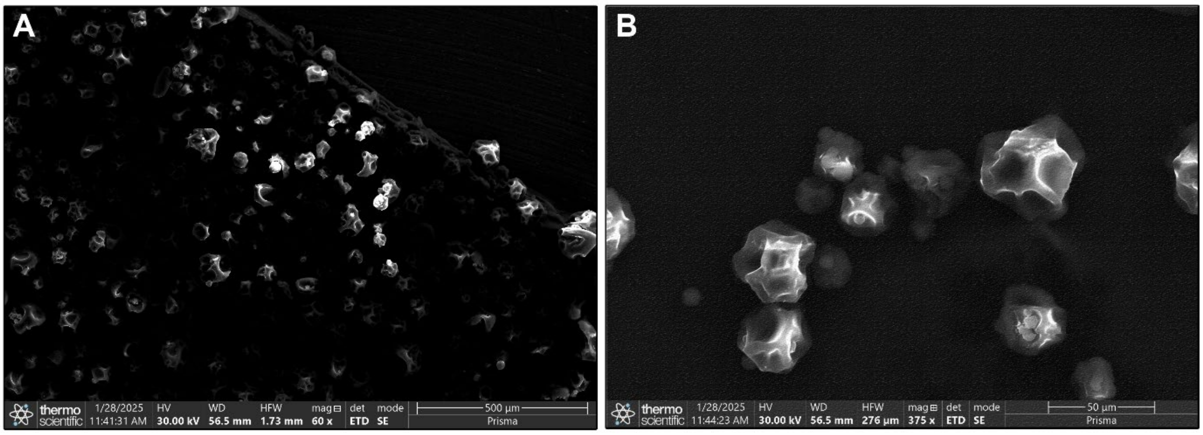

Disclosure of Potential Conflicts of Interest

The authors deny any conflicts related to this study.

Source of Funding: This study was supported by grants from the Conselho Nacional de Desenvolvimento Científico e Tecnológico (CNPq), Coordenação de Aperfeiçoamento de Pessoal de Nível Superior (CAPES), the Fundação de Amparo à Pesquisa do Estado de Minas Gerais (FAPEMIG). Dr. Guimarães, Dr. Santos, and Dr. de Paula are research fellows of the CNPq. Dra Farias is a research fellow of FAPEMIG.

Acknowledgments

The authors express their sincere gratitude to the Multiuser Laboratories for their invaluable support in conducting this research. We extend our appreciation to the Biotério de Criação e Experimentação Animal, as well as to the Laboratório de Microscopia Avançada for their expertise and technical assistance in imaging analysis. We also acknowledge the contributions of the Laboratório de Imagiologia Experimental in Vivo for their support in experimental imaging. Additionally, we are grateful to CITOMOL - Laboratório de Citometria, Cultivo Celular e Biologia Molecular for their support in cell culture and molecular biology experiments. This research would not have been possible without the infrastructure, resources, and technical expertise provided by these institutions.
